# Fast Sex Wellness Products Screening Using a Portable/Field Deployable MS Equipped with a Direct Sampling Atmospheric Pressure (DSAP) Source

**DOI:** 10.1021/jasms.6c00317

**Published:** 2026-10-07

**Authors:** Alberto Asteggiano, Alice Di Bernardo, Claudio Medana, Enrico Davoli

**Affiliations:** Department of Molecular Biotechnology and Health Sciences, 547618 University of Turin, Piazza Nizza 44, Torino 10126, Italy

**Keywords:** portable mass spectrometry (PMS), sex wellness products, adulteration analysis, PDE5i, DSAP MS

## Abstract

Sex-wellness products, including herbal formulations, API-containing tablets, creams, and sprays, are widely used to enhance sexual performance, treat erectile dysfunction, or improve perceived sexual well-being. These products span a broad and heterogeneous market, ranging from regulated prescription pharmaceuticals to over-the-counter (OTC) preparations and illicit or counterfeit (with APIs) products distributed through informal channels and online platforms. Particularly, the circulation of counterfeit, adulterated, or products marketed as alternatives to phosphodiesterase type 5 inhibitors (PDE5i), represents a growing public health challenge, where PDE5i and certain antidepressants and anesthetics used for the treatment of premature ejaculation are often detected in unregulated formulations. Current analytical approaches for illegal substances in these formulations rely predominantly on laboratory-based techniques, such as LC-HRMS/MS, which require time-consuming sample preparation, skilled personnel, and centralized facilities. Faster and cheaper screening approaches, such as immunoassays (e.g., ELISA) and other rapid test kits, widely used due to their simplicity and low cost, are inherently target-specific and may suffer from cross-reactivity or limited selectivity. Portable mass spectrometry (PMS) instrumentation represents an effective alternative, enabling rapid, on-site screening of complex samples with enhanced selectivity and broader detection capabilities. In this work, a miniature ion trap system, equipped with atmospheric pressure chemical ionization (APCI) and thermal desorption (TD), enabling MS and MS/MS analyses with minimal sample preparation, was evaluated for the rapid screening of pharmaceutical compounds in sex-wellness products, including counterfeit and adulterated formulations. Besides qualitative identification, a semiquantitative analysis was also developed to estimate drug concentration. A complementary LC-HRMS/MS analysis provided confirmation and structural elucidation of the compounds detected during on-site screening. The PMS instrumentation provided rapid and reliable screening and semiquantitative capabilities with results consistent with those obtained by LC-HRMS/MS systems.

## Graphical abstract

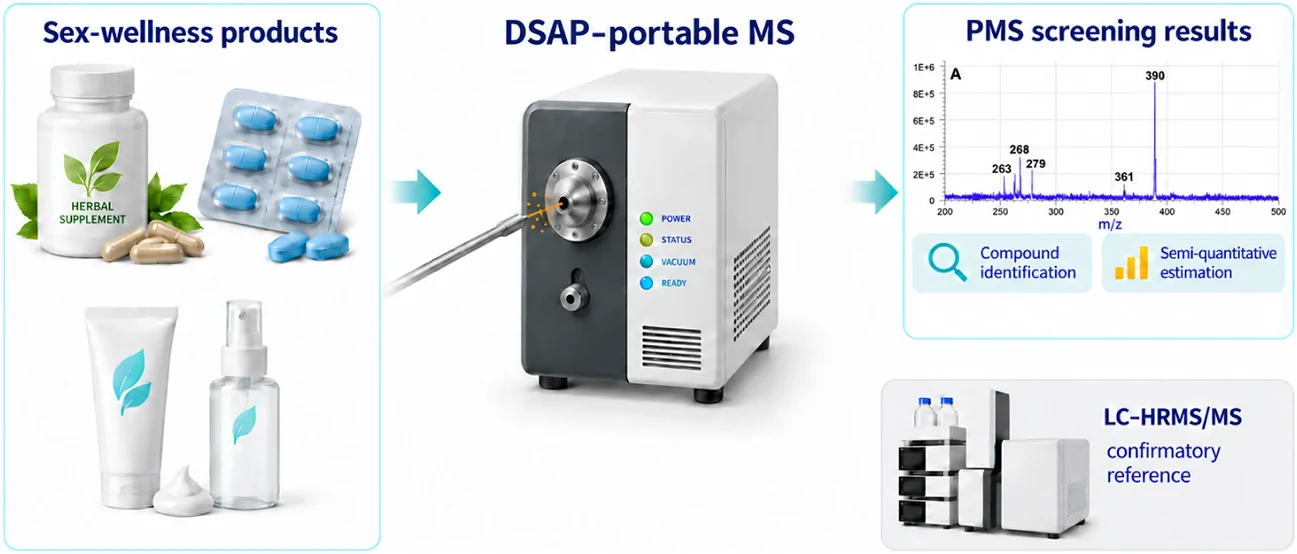

## Introduction

It is now well accepted that self-medication plays a major role in the healthcare system. This point has been supported by the realization that people are ultimately responsible for their own health and that seeking medical attention for minor illnesses is frequently not essential.^1^ In recent years, the global market has experienced a significant expansion not only in conventional OTC products but also in sex wellness supplements and herbal formulations marketed as “natural” remedies, which are particularly perceived as safer or culturally more accepted.^2^ Products purchased for sexual wellness, even when categorized as self-medication, are not consistently chosen with full awareness, and it is noteworthy that a part of consumers of these products actively seek counterfeit medications or formulations containing active ingredients that typically necessitate a physician’s prescription. There is indeed evidence indicating misuse of these products among younger people and their use by older individuals without prior consultation with their primary healthcare provider.^3,4^ Men predominantly seek remedies for addressing erectile dysfunction or premature ejaculation, which are prevalent male sexual dysfunctions.^5^ Treatment options generally involve active pharmaceutical ingredients that require a medical prescription. These substances primarily fall into two categories: phosphodiesterase 5 inhibitors (PDE5i) for erectile dysfunction^6–8^ and predominantly tricyclic antidepressants or selective serotonin reuptake inhibitors for premature ejaculation.^9^ Commercial products associated with this active ingredient are now well known among the general population, and their widespread adoption as lifestyle drugs through media and social networks has attracted the attention of counterfeiters.^4,10^ These products are even sold at very low prices in countries where drug pricing is not affected by patent protection or regulatory frameworks governing intellectual property rights and positioned as affordable substitutes for prescription therapies, further increasing their diffusion and potential for inappropriate or uninformed use. The problem is that products of this nature can be counterfeited, contaminated with other drugs, or have incorrect dosages, which raises serious concerns about indiscriminate use. In this scenario, the issues with counterfeit drugs cannot be underestimated. It is a growing public health problem, and despite various alerts being raised to the attention of authorities, the market continues to expand.

In parallel, increasing attention has been directed toward so-called “natural” or herbal supplements marketed for sexual enhancement, which are often perceived by consumers as safer alternatives to conventional pharmacological therapies. However, a substantial body of evidence has demonstrated that many of these products are intentionally adulterated with undeclared active pharmaceutical ingredients, particularly phosphodiesterase 5 inhibitors (PDE5i) or their structural analogues.^11^ These compounds are frequently added to enhance efficacy while avoiding regulatory scrutiny, as the products are sold under the guise of dietary supplements or traditional remedies.^2,12^

The detection of undeclared pharmaceutical ingredients in these complex matrices represents a significant analytical challenge.^13^ Herbal supplements are characterized by highly variable and heterogeneous compositions, often containing a wide range of natural compounds that can interfere with measurements, while unofficial formulations might contain drug analogues or multiple active substances. This screening challenge requires modern, sensitive, and selective techniques; among these, immunoassay-based techniques, such as enzyme-linked immunosorbent assays (ELISAs), have been widely employed as rapid screening tools due to their simplicity, relatively low cost, and high throughput.^14^ Nevertheless, these methods are inherently limited by issues of cross-reactivity, restricted selectivity toward structurally similar analogues, and the inability to provide a definitive structural identification of unknown compounds of multiple molecular classes. Furthermore, compared to MS-based analytical methods, ELISAs may provide lower sensitivity depending on antibody affinity, matrix composition, and assay configuration.^15–17^

For these reasons, LC-MS/MS-based techniques are considered the gold standard for the identification and quantification of pharmaceutical drugs and adulterants, owing to their high sensitivity, specificity, and capability to detect multiple compounds simultaneously.^13,18^ Despite these advantages, such techniques typically require extensive sample preparation, well-equipped laboratory facilities, and trained personnel, resulting in longer analysis times and limited applicability for rapid on-site screening.

These limitations highlight the need for analytical strategies that combine the rapid response and operational simplicity of screening methods with the selectivity and reliability of confirmatory techniques.

Although PMS use has been increasingly investigated for forensic, environmental, and security applications, its use for the analysis of counterfeit and adulterated sex-wellness products remains very limited. Most analytical studies addressing these products have relied on laboratory-based LC-MS methodologies, whereas the analytical performance of portable MS systems for rapid field screening of these highly heterogeneous matrices has not yet been systematically evaluated. Furthermore, very few studies have assessed the capability of miniature MS instruments to provide both structural confirmation through MS/MS experiments and semiquantitative information under field conditions.

In this context, PMS has emerged as a promising solution, offering the possibility of direct, *in situ* analysis with minimal sample preparation while maintaining high analytical performance.^19,20^ For these reasons, we evaluated the effectiveness of a new portable mass spectrometer—the MassTech MTE30—in analyzing medicinal products, counterfeit alternatives, and natural products purchased from local street markets, retail street vendors, international markets, global distribution networks, or coming from police seizures. The objective was to provide reliable and rapid responses within a very short time frame.

The PMS used is a fully integrated portable mass spectrometry platform weighing approximately 17 kg, specifically designed for on-site applications, enabling analyses to be performed directly in retail environments, street markets, distribution channels, and points of inspection, such as customs or law enforcement seizures, while ensuring rapid response and user-friendly operation. The system is based on a miniature ion trap mass spectrometer featuring a wide mass range and the capability to perform both MS and MS/MS analyses.

The instrument is equipped with an APCI source coupled to a TD unit, allowing for direct analysis of samples with minimal preparation. In fact, sample handling is limited to a simple dilution in solvent or extraction for nonsoluble samples like herbal or tablet products, after which a small aliquot is thermally desorbed and introduced into the APCI source for ionization and subsequent mass spectrometric analysis.

The aim of this study was therefore to evaluate the analytical performance of a new miniature APCI–thermal-desorption ion trap mass spectrometer for the rapid screening of pharmaceutical compounds in counterfeit medicines, herbal supplements, and other sex-wellness products. Compared to previous studies relying exclusively on laboratory-grade instrumentation, the present work assesses the capability of a PMS platform to perform direct MS and MS/MS analyses with minimal sample preparation, providing analytical results comparable to those obtained by reference LC-HRMS/MS methods. In addition, this study demonstrates the feasibility of obtaining reliable semiquantitative information when used in targeted mode, extending the role of PMS from qualitative screening to a more informative on-site analysis. To the best of our knowledge, this study represents one of the first comprehensive evaluations of a miniature APCI portable ion trap mass spectrometer for the forensic direct analysis of adulterated sex-wellness products in highly heterogeneous and analytically challenging matrices, including herbal formulations, tablets, creams, and sprays.

This semiquantitative approach proves to be extremely useful in the identification of pharmacologically active compounds, given the need to observe specific dosages of the detected molecules.

## Materials and Methods

### Chemicals and Reagents

Lidocaine, sildenafil, tadalafil, vardenafil, avanafil, dapoxetine, and formic acid (98–100%) were purchased from Sigma-Merck (Milan, Italy). Sildenafil-d3, used as an internal standard for the semiquantitative analysis, was purchased from MedChemExpress (Monmouth Junction, NJ, USA). Methanol (MeOH) and acetonitrile (ACN) (LC–MS grade) were from VWR (Milan, Italy). For the preparation of standard stock solutions at a concentration of 1000 mg/L, all the reference standard compounds were solubilized in methanol and diluted in the final solution of analysis (for PMS) or starting chromatographic eluent (for LC-HRMS/MS). All aqueous solutions were prepared with HPLC-grade water from a Milli-Q Academic System (Millipore, Burlington, MA, USA).

### Sample Collection and Preparation

Samples including creams, pills, sprays, food supplements, powders, and herbal products, were purchased from local street markets and drugstores, or were provided out of police seizures. Medicines in tablet form were purchased from street vendors or pharmacies. Table S1 reports an overview of the samples analyzed. Once collected, samples were stored in the dark at room temperature until analysis, taking care to avoid direct sunlight or excessive temperature or humidity exposure. Tablets (after crushing), supplements, and cosmetic preparations were extracted by shaking the vial by hand with methanol, suspending 1 g of the product in 10 mL of solvent. This extraction step was chosen to better be suitable in on-field conditions. The supernatant solutions were filtered (45 μm) and diluted for unknown compounds analysis in methanol/Milli-Q water. Various dilution factors were tested to achieve the highest sensitivity during sample analysis using the PMS instrument. A semiquantitative analysis was performed by spiking selected sildenafil-containing samples with a known concentration of sildenafil-d_3_ standard as IS. The semiquantitation was achieved by area comparison with the IS. For the analysis, 2 μL of each diluted sample was directly placed on the disposable glass plate over the IR illuminator on the DSAP ion source of the instrument, starting from the highest dilution to the lowest to prevent instrumental contamination. For LC-HRMS/MS confirmatory analysis, solid samples (tablets, capsules, and creams) were weighed (∼100 mg) and extracted with 10 mL of MeOH using ultrasonic-assisted extraction, maintaining a bath temperature <30 °C. The resulting extracts were separated from the insoluble fraction by centrifugation (10000*g,* 10 min, RT), and the supernatants were then subjected to a 10,000-fold dilution with the initial mobile phase prior to HPLC-HRMS analysis. Liquid samples were aliquoted (10 μL) and diluted in 1 mL of MeOH, after this step the solution was centrifuged to separate all the insoluble components, and the supernatants were diluted 100-fold with the initial mobile phase prior to HPLC–HRMS analysis.

#### PMS Instrumentation

Experiments were conducted using an MT Explorer 30 (MTE30, MassTech, Inc., Columbia, MD, USA) coupled with a Direct Sampling Atmospheric Pressure (DSAP) source equipped with an APCI module. Data acquisition was performed in MS mode using proprietary MODAS software. Qualitative data analysis was carried out using ChromExplorer 1.5.1 software. For each MS analysis, the total scan duration was less than 1 s. Capillary temperature was set at 220 °C, spray voltage was kept at 2.4 kV, and thermal desorption was used, setting the IR illuminator at 50% power. MS was operated in full scan mode, in the range of *m*/*z* 50–800, with an injection time of 20 ms for MS analysis, and using automatic gain control (AGC) for MS/MS experiments. For MS/MS experiments, the precursor ion isolation was 2 Da, the Q factor was kept at 0.25, and the fragmentation energy was compound-dependent, varying from 10% to 90%. The instrument was factory-calibrated and, after shipment to the lab, the calibration was daily checked with caffeine and reserpine reference standards, and recalibration was never needed, being within the manufacturer specification.

#### LC-HRMS/MS for Confirmatory Analysis

Separation and analysis for compound confirmation were performed using a Thermo Vanquish Core liquid chromatograph coupled through an ESI source to an Orbitrap Exploris 120 mass spectrometer (Thermo Scientific, Milan, Italy).

HPLC separation was obtained using, as stationary phase, a reverse phase column (Luna C18(2), 150 × 2.1 mm, 3 μm; Phenomenex, Castel Maggiore, BO, Italy) and, as mobile phase, 0.1% formic acid in water (A) and acetonitrile (B). The gradient elution ramp was as follows: 0–1 min, 10% B, 100% B in 19 min, held 2 min, and restored to the initial conditions, for a total run time of 26 min. Nitrogen was used as auxiliary and sheath gases for the ESI source, working in positive-ionization mode. The HRMS parameters were set as follows: sheath gas 35 arbitrary units (arb), auxiliary gas 20 arb, capillary voltage 3.5 kV in positive ion mode, ion-transfer tube temperature 325 °C. For each sample, two different acquisition modes were performed: full-scan MS (FS) and data-dependent MS^2^ (ddMS^2^) scan. Full-scan was performed in the range 50–800 *m*/*z* with R = 45K, and ddMS^2^ acquisition mode using HCD 30%. MS^2^ spectra were acquired with R= 22.5K. For the confirmation of drug structures, the software Xcalibur 4.0.27.13 (Thermo Scientific), based on mass accuracy (<5 ppm) and ring and double-bond (RDB) values, was used. To achieve level 1 identification level^21^ we compared retention times and MS^2^ spectra of the unknowns with the corresponding standard compounds. Level 2 was achieved for the putative compounds through MS^2^ fragmentation patterns, and MS^1^ formula prediction by accurate mass with Δppm <5.

## Results

### Instrumental Performance

For the PDE5i included in the study—sildenafil, tadalafil, vardenafil, avanafil—as well as for dapoxetine and lidocaine, the portable system consistently produced protonated molecular ions [M + H]^+^ in good agreement with the expected nominal masses, then confirmed by HRMS. Sildenafil was detected at *m*/*z* 475 (HRMS-accurate monoisotopic mass: 475.2118 *m*/*z*, theoretical monoisotopic mass: 475.2120 *m*/*z*), tadalafil at *m*/*z* 389 (HRMS: 390.1448 *m*/*z*, theoretical: 390.1445 *m*/*z*), vardenafil at *m*/*z* 489 (HRMS: 489.2276 *m*/*z*, theoretical: 489.2278 *m*/*z*), and avanafil at *m*/*z* 484 (HRMS: 280 484.1856 *m*/*z*, theoretical: 484.1859 *m*/*z*), all consistent with the expected molecular weights of the respective APIs. Dapoxetine, an SSRI drug, showed a molecular ion at 306, all consistent with the expected molecular weights of the respective APIs. Dapoxetine, an SSRI drug, showed a molecular ion at 306 *m*/*z* (theoretical monoisotopic mass: 306.1852 *m*/*z*) while the local anesthetic lidocaine registered a 235 *m*/*z* signal against a theoretical monoisotopic mass of 235.1804 and an HRMS value of 235.1794 *m*/*z*.

Complementary analysis by LC-HRMS/MS was performed on all of the standards to provide confirmatory identification and accurate formula characterization of the detected compounds. The HRMS measurements yielded accurate masses in agreement with the theoretical monoisotopic values for all seven analytes, with mass errors spanning from −3.4 ppm (lidocaine) to +0.51 ppm (tadalafil).

In addition to molecular ion detection, the portable system provided MS/MS fragmentation data, yielding low-resolution product ion spectra that recapitulated the main fragmentation pathways of each molecule. For instance, for sildenafil, fragment ions were observed at *m*/*z* 447, 432, 377, 311, and 283, corresponding to well-characterized losses from the piperazine-sulfonylethyl moieties confirmed by HRMS/MS as reported^22^ (Figure 1). Tadalafil produced fragments at *m*/*z* values of 279, 268, 263, and 262, corresponding to the observed HRMS/MS 268.1081 and 262.0862 *m*/*z* ions. Vardenafil yielded a fragmentation spectrum with *m*/*z* values of 376, 344, and 299, reflected in the HRMS/MS spectrum for the ions 376.1071 299.1137 *m*/*z*. Avanafil, in PMS-MS/MS analysis, yielded *m*/*z* 375 and 349 fragment ions whose presence was confirmed by HRMS/MS 375.1221, 349.1227 *m*/*z*.

**Figure 1:**
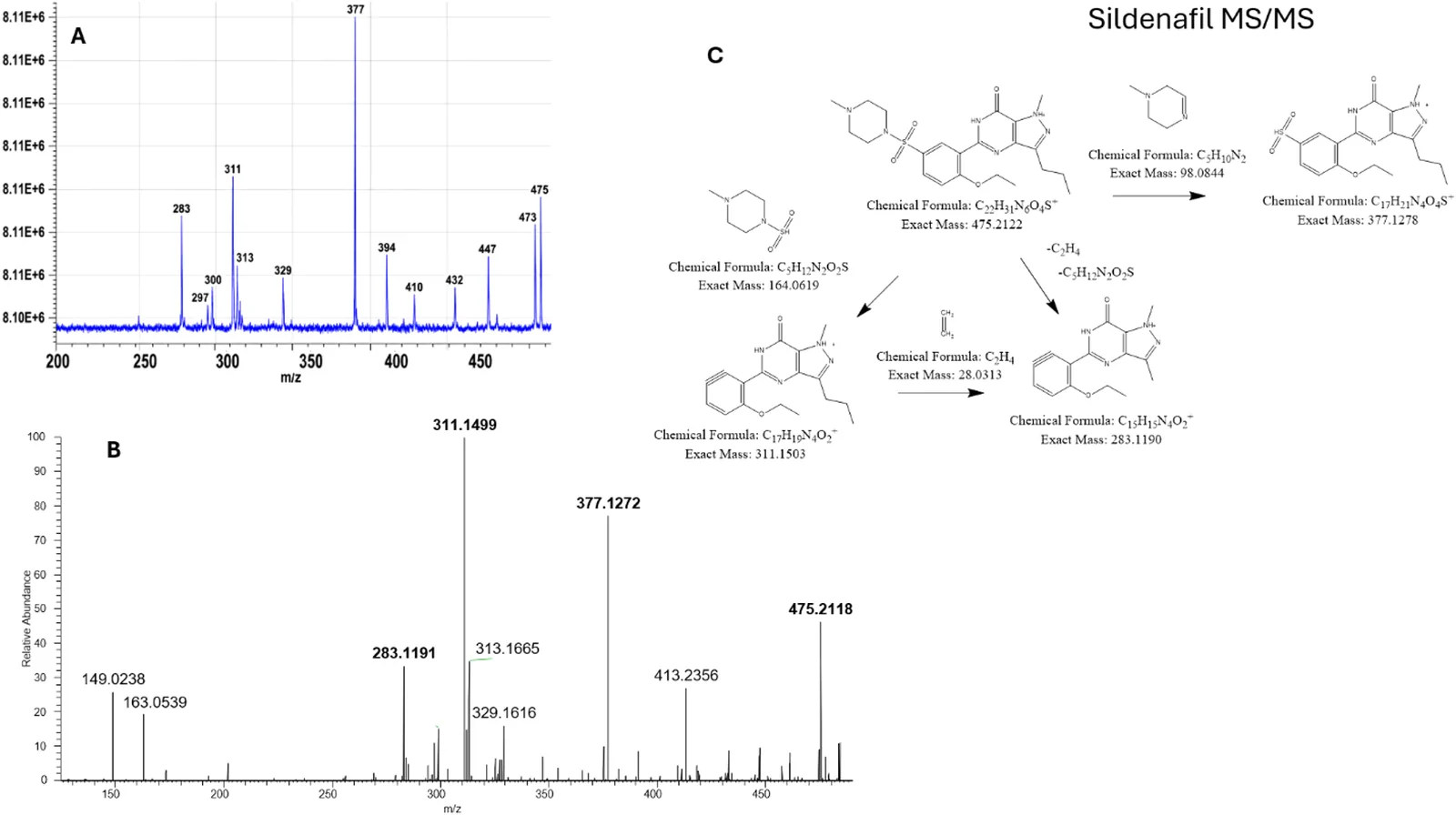
Sildenafil MS/MS spectra obtained by PMS (A), HRMS (B), and sildenafil’s proposed fragmentation pattern (C).

Lidocaine fragmentation showed a unique ion at 86 *m*/*z*, whose presence was confirmed by HRMS at 86.0961 *m*/*z*. Fragmentation of dapoxetine yielded characteristic product ions at *m*/*z* 261, 183, 163, and 149 in MS/MS experiments. These fragments were further validated by HRMS/MS, with ions at *m*/*z* 261.1272 and 183.0802. These MS/MS fingerprints, although at unit resolution, provide a first layer for portable forensic and putative identification applications; Table 1 shows all the MS^1^, MS/MS values of ions’ *m*/*z*. PMS-to-HRMS-compared fragmentation patterns of tadalafil, vardenafil, avanafil, and dapoxetine are reported in Supplementary Figures S3–S6.

**Table 1:** Annotated Molecules and Their Annotation Level.^a^

		PMS		HRMS	
Molecule (confidence level)	[M + H]^+^ theoretical (*m*/*z*)	[M + H]^+^measured (*m*/*z*)	MS/MS main fragments (*m*/*z*)	MS measured(*m*/*z*), (Δppm)	LC-HRMS/MS main fragments (*m*/*z*)
sildenafil (1)	475.2120	475	**447**, 432, **377**, 311, **283**	475.2118, (−0.42)	447.1810, 377.1272, 311.1499, 283.1191
tadalafil (1)	390.1445	389	279, **268**, 263, **262**	390.1446, (−0.51)	268.1081, 262.0862, 250.084, 135.0441
vardenafil (1)	489.2278	489	**377**, 344, **299**	489.2276, (−0.41)	376.1071, 339.1482, 312.1578, 299.1137
avanafil (1)	484.1859	484	**375, 350**	484.1856, (−0.72)	375.1221, 349.1227
dapoxetine (1)	306.1852	306	**261**, **183**, 163, 149	306.1851, (−0.33)	261.1272, 183.0802, 117.0698
lidocaine (1)	235.1804 (+)	235	**86**	235.1794, (−3.4)	86.0961, 58.0684 (+)
hydroxyhomosildenafil (2)	505.2227	505	**487**, **377**	505.2222, (−1.18)	487.2118, 461.1961, 377.1270, 311.1502, 283.1184

a The second column shows the theoretical *m*/*z* value in HRMS. The third and fourth column show the obtained MS^1^ and MS^2^ signals through PMS while fifth column the MS^1^ value obtained through HRMS analysis and the accuracy in ppm against the theoretical *m*/*z*, and the sixth column reports the accurate MS/MS values.

### Sample Analysis

All labeled prescriptible formulations including well-known products such as Viagra, Levitra, and Spedra—showed qualitative agreement with their API compositions. This consistency not only confirms product authenticity but also indirectly supports the reliability of the analytical workflow, as both the portable MS and HPLC-HRMS/MS approaches converged on the same identifications, with coherent fragmentation patterns also comparable to standard molecules (see Table 2).

**Table 2:** Qualitative Analytical Results Obtained with PMS Analysis

Sample Name	Sample Form	Active Principle Declared	Active Principle Identified
Spedra	Tablet	avanafil 200 mg	as declared
Cialis	Tablet	tadalafil 20 mg	as declared
Levitra	Tablet	vardenafil 20 mg	as declared
Viagra	Tablet	sildenafil 50 mg	as declared
Joyolgra	Tablet	sildenafil citrate 100 mg	as declared
Vigore 100	Tablet	sildenafil citrate 100 mg	as declared
Suhagra 100	Tablet	sildenafil citrate 100 mg	as declared
Viro 100	Tablet	sildenafil citrate 100 mg	as declared
Cenforce 100	Tablet	sildenafil citrate 100 mg	as declared
Caverta	Tablet	sildenafil citrate 100 mg	as declared
Suhagra 50	Tablet	sildenafil citrate 50 mg	as declared
Apcalis-SX 20	Tablet	tadalafil 20 mg	as declared
Super Kamagra	Tablet	sildenafil citrate 100 mg dapoxetine citrate 60 mg	as declared
Lovegra	Tablet	sildenafil citrate 100 mg	as declared
Vigas 50000	Spray	None	lidocaine
ViaMax Power	Herbal product (tea)	None	sildenafil
Big Bang	Cream	None	lidocaine
Develope sex	Cream	None	lidocaine
Jekonmo	Herbal product, Liquid	None	sildenafil
Manisa	Herbal product, Liquid	None	sildenafil
Mudet	Herbal product, Liquid	None	sildenafil
Ceedra	Herbal product, Capsule	None	hydroxyhomosildenafil (lodenafil)
Street market blue pills	Tablet	None	none
Street market white pills	Tablet	None	none

Generic formulations purchased from Indian and Thai street markets were likewise confirmed to contain sildenafil and tadalafil. Multiple active ingredients were concurrently detected in combination products. Particularly, the case of Super Kamagra, a tablet containing sildenafil and dapoxetine (an SSRI used in the control of premature ejaculation), confirmed the capability of speed and specificity of this PMS approach (as shown in Figure 2Aand [Fig fig2]B), also in this case, LC-HRMS/MS analysis confirmed the obtained results.

**Figure 2:**
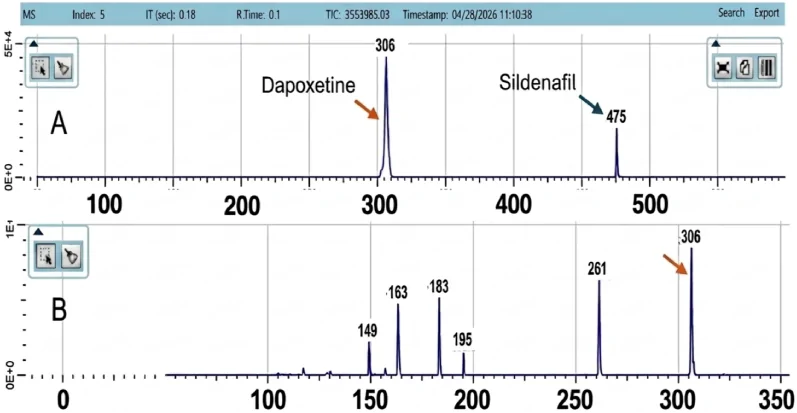
(A) shows the PMS MS^1^spectrum for Super Kamagra combined formulation; ions of 306 and 475 *m*/*z*, namely dapoxetine and sildenafil, are reported; (B) shows the MS^2^spectrum of dapoxetine.

In contrast, several products marketed as “herbal” or without declared active compounds were found to contain pharmacologically active substances. Indeed, sildenafil was identified in multiple samples (e.g., ViaMax Power, Jekonmo, Manisa, Mudet), while topical and spray formulations (Vigas 50000, Big Bang, Develop Sex) were consistently found to contain lidocaine. These identifications were achieved at confidence level 1 ^21^ through comparison of retention times and MS/MS spectra with reference standards, and confirmed through LC-HRMS/MS analysis.

Notably, two unpackaged generic tablet samples did not yield detectable signals attributable to known active pharmaceutical ingredients in either the screening or confirmatory analysis.

Overall, the combined use of PMS and HPLC–HRMS/MS provided a robust and complementary strategy, enabling rapid screening followed by high-confidence structural confirmation, and revealing systematic adulteration in nonregulated products.

### Annotation of Hydroxyhomosildenafil, a Sildenafil Analogue

A signal at 505 *m*/*z* was found as the main component of the Ceedra herbal product, an encapsulated powder formulation resulting from a police seizure in Italy. The MS/MS spectrum showed signals at 487, 377, and 162 *m*/*z* (Figure 3). The signal was then compared with the one generated by LC-HRMS/MS untargeted analysis, and the high-resolution-based fragmentation study proposed its putative identification as hydroxyhomosildenafil (lodenafil). HRMS measurement yielded an accurate mass of *m*/*z* 505.2221 against a theoretical value of 505.2227 (Δ = −1.19 ppm). The HRMS/MS fragmentation profile showed ions at *m*/*z* 487.2118 (loss of water), 461.1961, 377.1270, 311.1502, and 283.1184. The fragments at *m*/*z* 377.1270, 311.1502, and 283.1184 are shared with those of sildenafil, reflecting the conserved scaffold common to both molecules.

**Figure 3:**
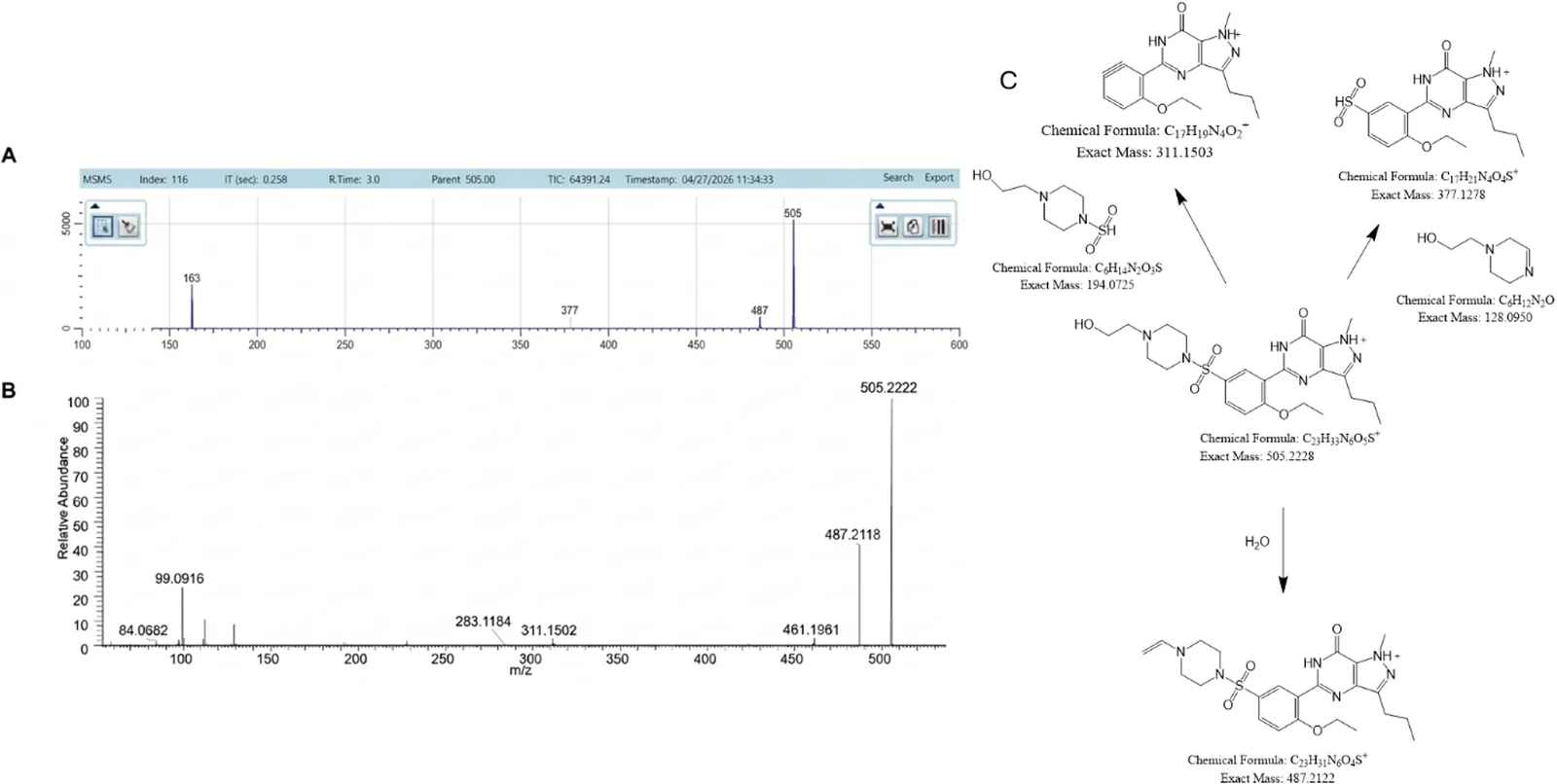
PMS (A) and HRMS/MS (B) spectra, and the relative fragmentation pattern of hydroxyhomosildenafil (lodenafil), are shown in the Ceedra sample.

Hydroxyhomosildenafil (lodenafil) detection in sex-wellness products is not unprecedented, since sildenafil analogues are among the most frequently reported undeclared adulterants in dietary supplements and herbal products marketed for sexual performance enhancement, particularly through unregulated online channels.^13^

### Semiquantitative Analysis of Sildenafil

The semiquantitative performance of the portable MS system was evaluated using sildenafil-d_3_ as an isotopically labeled internal standard. The sildenafil-to-sildenafil-d_3_ signal ratio increased proportionally with the corresponding concentration ratio over the investigated range of 0.2–5.0 μg/mL, with an R^2^ value of 0.98, n = 3. Accuracy and precision have been evaluated through the study of BIAS% and RSD% showing values exceeding 25% only for BIAS% in the lowest calibration point (Figure S2).

The estimated sildenafil content ranged from 0.04 to 25.94% w/w. Super Kamagra showed the highest estimated concentration (25.94% w/w; RSD = 3.07%, *n* = 3), followed by Lovegra (16.67%w/w; RSD = 9.06%). Lower concentrations were estimated for liquid samples: Jekonmo (5.20% w/w; RSD = 21.11%), Manisa (0.90% w/w; RSD = 11.16%), and Mudet (0.04% w/w; RSD = 25.63%). All results are shown in Tables 3. Tablet formulations found in informal markets, such as Lovegra and Super Kamagra, showed results in strong accordance with the labeled declared dosage with good intrasample variability with detected abundance/declared dose % of namely 106.9 and 100.2. Liquid formulations such as Manisa, Jekonmo, and Mudet registered lower repeatability, but always in the <26% RSD range. Ceedra showed a hydroxyhomosildenafil concentration of 15.92 w/w, RSD = 46.29%. This semiquantitative estimation showed the highest variability among replicates. This variability can be related to the heterogeneous nature of the encapsulated sample, which appears coarse and grainy.

**Table 3:** Quantitative Analytical Results Obtained with PSM

Sample name	Sildenafil extimated by PMS (% w/w); RSD%, *n* = 3	Detected abundance/declared dose (%)
Super Kamagra	25.94; 3.07	106.9
Jekonmo	5.20; 21.11	**not declared**
Mudet	0.04; 25.63	**not declared**
Manisa	0.90; 11.16	**not declared**
Lovegra	16.67; 9.06	100.2
Ceedra	15.92; 46.29^a^	**not declared**

a Hydroxyhomosildenafil expressed as sildenafil-d3.

## Discussion

The role of the PMS investigated in this study should be considered in the context of a tiered analytical workflow in which rapid field screening and laboratory-based confirmatory analysis serve complementary purposes. The PMS is intended to provide rapid, qualitative, and semiquantitative on-site information for the detection and prioritization of potentially adulterated samples.

In this work, the use of a field-deployable ion-trap mass spectrometer with an atmospheric pressure interface capable of APCI led to the detection of several active compounds in sex wellness products. A key objective of this study was to evaluate the degree of concordance between the on-site portable ion-trap screening results and the laboratory-based LC-HRMS/MS confirmatory data. For sildenafil, tadalafil, vardenafil, avanafil, dapoxetine, and lidocaine, the nominal masses recorded by the portable instrument were in full agreement (with MS accuracy of >0.5 *m*/*z*) with the accurate masses subsequently measured by HRMS, with no false positives or misassignments observed in sample analysis. Most samples were acquired in the laboratory under relatively controlled environmental conditions; however, the PMS has been moved and widely used in different laboratories for the analysis of different drug seizures. Therefore, demonstrating consistency with reference instrumentation represents important confirmation of analytical reliability.

The MS/MS fragmentation fingerprints recorded at unit resolution by the portable system were consistent—by sharing the highest abundant peaks—with the HRMS/MS analysis, confirming that the fragmentation pathways are reproducible for most of the ions across the two platforms despite differences in ionization source and ion trap geometry. The MS/MS capability of this PMS also guarantees a further level of confidence in analyte detection and confirmation, thus increasing analysis specificity.

These findings demonstrate that the miniaturized APCI-TD ion trap system is capable of reliably detecting the principal pharmacologically active substances in sex-wellness products at concentrations relevant to real-world formulations and that its results are confirmable by laboratory LC-HRMS/MS without contradiction. The analytical capabilities of the approach were first assessed by detecting declared APIs in branded formulations such as Levitra, Cialis, Viagra, and Spedra. Then, a series of samples coming from informal street markets with labeled APIs (mimicking the well-known branded sexual wellness products) showed the presence of the same active ingredients (PDE5i and SSRI drugs). Sildenafil and tadalafil were indeed found to be present in Indian and Thai informal street-market- sold formulations such as Joyolgra, Vigore 100, Suhagra 100, Viro 100, Cenforce 100, Caverta, Suhagra 50, Apcalis-SX 20, Super Kamagra (which concurrently showed the presence of Dapoxetine) and Lovegra (see Table S1). The findings of APIs in similar formulations have been widely documented.^23^ A complementary semiquantitative approach has also been integrated and evaluated with BIAS% and RSD% performance indicators on a set of selected samples; from a forensic and regulatory perspective, semiquantitative concentration information adds practical value to qualitative identification. A positive result establishes the presence of an active compound, whereas a semiquantitative estimate can distinguish samples containing low, intermediate, or high analyte levels and prioritize those requiring urgent confirmatory quantification or toxicological assessment. In this view, this system has been able to accurately estimate APIs from a binary presence/absence and abundance perspective toward an analytical decision-support system for field triage. The results pose that PMS-based triaging is indeed as a time-saving methodology that offers a clear advantage over traditional laboratory-based techniques in terms of execution speed, while offering greater specificity and flexibility than conventional ELISA-based screening approaches. On the other hand, it does not provide the same level of quantitative robustness and analytical certainty as laboratory-based instrumental methods, which are required for final positivity confirmation.

The medical issues of counterfeit drugs and natural products are—besides the well-known grossly incorrect dosages, contaminants, or adulterants—also given by the presence of analogs,^4^ as found in the Ceedra sample, whose analysis allowed the annotation at second level^21^ of a sildenafil analogue: hydroxyhomosildenafil.^24,25^ Additionally, the approach showed the complete absence of active principles in two different samples. The finding has been confirmed by HRMS analysis. Alongside PDE5i, local anesthetics such as lidocaine were consistently identified in topical and spray formulations.^26^ This result exemplifies how different classes of active compounds are deliberately incorporated to achieve specific functional effects—erectile enhancement in the case of PDE5i, and modulation of ejaculation latency in the case of lidocaine—while remaining undeclared to the consumer.^7^

From a toxicological and public health perspective, the presence of undeclared pharmacologically active substances or the ease of finding active substances without medical prescription represents a significant risk. The uncontrolled intake of PDE5i may lead to adverse cardiovascular events, particularly in individuals with underlying conditions or in combination with contraindicated medications, while the presence of analogues further increases uncertainty in terms of pharmacokinetics and toxicity.

Lidocaine in creams, lotions, and associated formulations for increasing intravaginal ejaculation latency time (IELT)^26,27^ is associated with side effects including delayed ejaculation (>30 min), reduced penile sensitivity, irritation, and decreased vaginal sensitivity, and its use is contraindicated in sensitized individuals.^28^ Furthermore, lidocaine has been linked to severe adverse events, including central nervous system toxicity following topical application, cardiac complications such as bradycardia, and, in rare cases, cardiac arrest and death even at therapeutic doses.^29–31^ Reports of adverse effects following the use of lidocaine-based sprays for sexual purposes further highlight the risks associated with their unregulated use.

Beyond the analytical results presented, these findings extend the potential applications of portable mass spectrometry. While previous applications have mainly focused on drugs of abuse, explosives, and security-related analyses, the present work demonstrates that a miniature APCI-TD ion trap platform can also be successfully applied to the screening of counterfeit and adulterated drugs, here, sex-wellness products, which represent highly heterogeneous and analytically challenging matrices. The capability of performing both MS and MS/MS analyses with minimal sample preparation, together with the additional semiquantitative information obtained for selected compounds, provides analytical results much better than conventional on-site screening approaches. These characteristics make the system particularly suitable in forensic, customs inspections, market surveillance, and rapid regulatory interventions, where timely analytical decisions are essential while confirmatory laboratory analyses are pending.

## Conclusions

The portable ion trap mass spectrometer proved to be a reliable tool for on-site screening and quantitative estimation of sex-wellness products, showing full agreement with laboratory LC-HRMS/MS results for all detected compounds. Consistent precursor ions and fragmentation patterns for the most abundant MS/MS ions, with no false positives, confirm its suitability as a first-tier analytical method, while HRMS provides definitive confirmation.

The detection of undeclared active substances, including PDE5i and lidocaine, highlights significant public health risks associated with these unregulated products. Given the rapid market growth and potential increase in adulteration, the integration of PMS for field screening with HRMS confirmation offers an effective strategy to support surveillance and regulatory controls.

## Supplementary Material



## Notes

The Supporting file provides, through a synopsis, additional data from analyzed samples; the semiquantitative calibration line, together with the main linearity and precision descriptors; and the proposed fragmentation pathways of the main drug analytes. The following files are available free of charge (PDF)

## ABBREVIATIONS

PMS: Portable Mass Spectrometer
APCI: Atmospheric Pressure Chemical Ionization
API: Active Pharmaceutical Ingredient
DSAP: Direct Sampling Atmospheric Pressure
ELISA: Enzyme-Linked Immunosorbent Assay
ESI: Electrospray Ionization
HCD: Higher-Energy Collisional Dissociation
HPLC: High-Performance Liquid Chromatography
HRMS: High-Resolution Mass Spectrometry
HRMS/MS: High-Resolution Tandem Mass Spectrometry
IELT: Intravaginal Ejaculation Latency Time
LC–MS/MS: Liquid Chromatography–Tandem Mass Spectrometry
MeOH: Methanol
MS: Mass Spectrometry
MS/MS: Tandem Mass Spectrometry
OTC: Over-The-Counter
PDE5: Phosphodiesterase Type 5
PDE5i: Phosphodiesterase Type 5 Inhibitors
PMS: Portable Mass Spectrometry
RDB: Ring Double Bond
SSRI: Selective Serotonin Reuptake Inhibitor
TD: Thermal Desorption
