# Supplementary material for: Fast Sex Wellness Products Screening Using a Portable/Field Deployable MS Equipped with a Direct Sampling Atmospheric Pressure (DSAP) Source
Source: J Am Soc Mass Spectrom. 2026 Oct 7;37(11):2906–14. doi: 10.1021/jasms.6c00317 (PMC13741922; doi:10.1021/jasms.6c00317)
Supplement: Supplementary file 1 [file js6c00317_si_001.pdf]

Supporting Information

Fast sex wellness products screening using a portable/field deployable MS equipped with a Direct Sampling Atmospheric Pressure (DSAP) source

Alberto Asteggiano *, Alice Di Bernardo , Claudio Medana , and Enrico Davoli

Department of Molecular Biotechnology and Health Sciences, University of Turin, Piazza Nizza 44, 10126, Torino, Italy

Corresponding author e-mail: alberto.asteggiano@unito.it

Figure S1: Semi-quantitative calibration curve (Sample sildenafil: Super Kamagra/Sildenafil-d_3)

[Sample/IS] µg/mL	Sample/IS peak high	Precision (RSD%)	Accuracy (BIAS%)
0.2	0.227	22.9	-32.4
0.5	0.384	9.37	-25.4
1	0.761	3.73	-3.04
2	1.683	5.54	17.5
5	3.099	8.55	-3.26

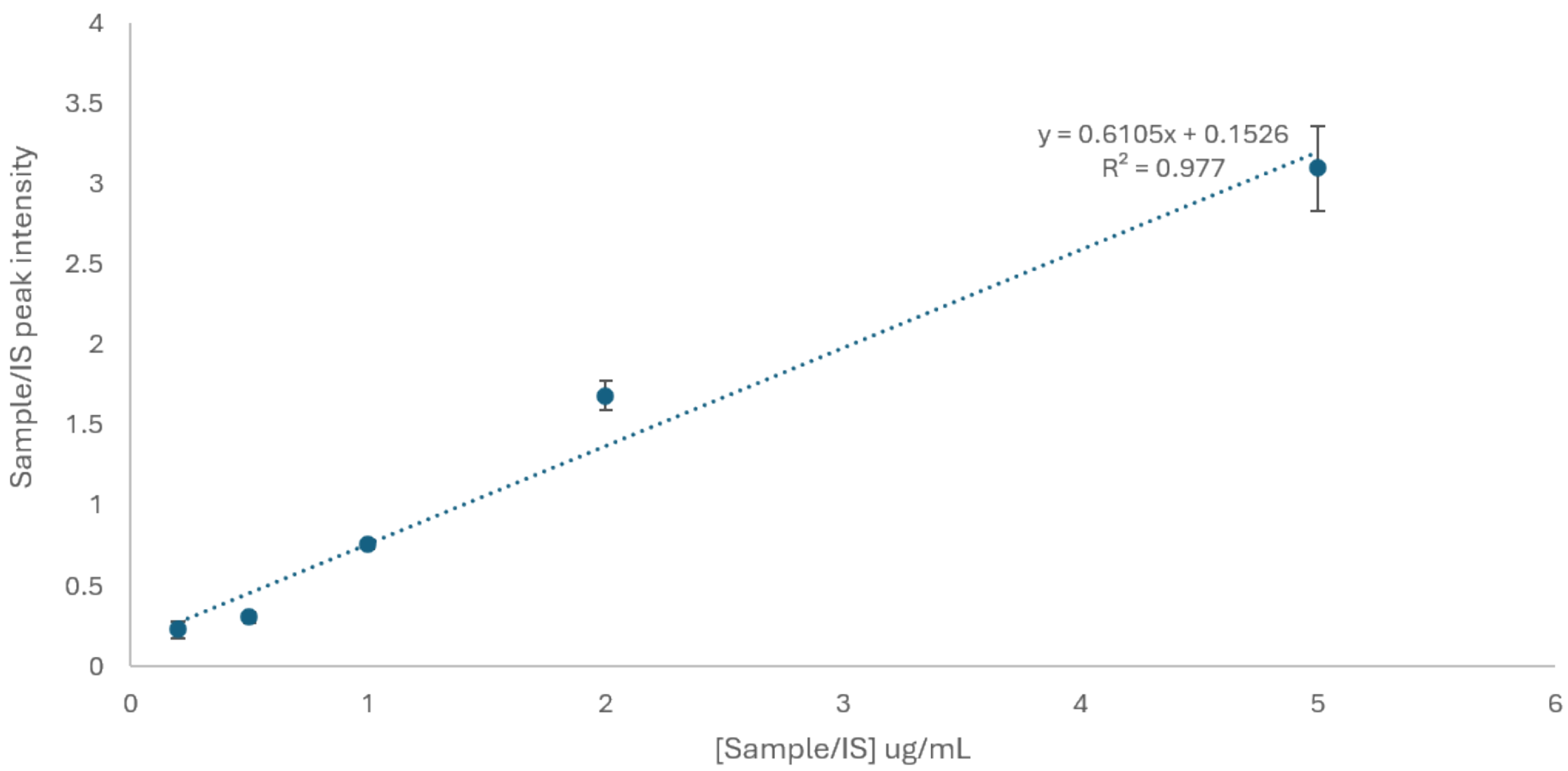

Figure S2: Lowest calibration level used for quantitation of Sildenafil and Sildenafil-d_3

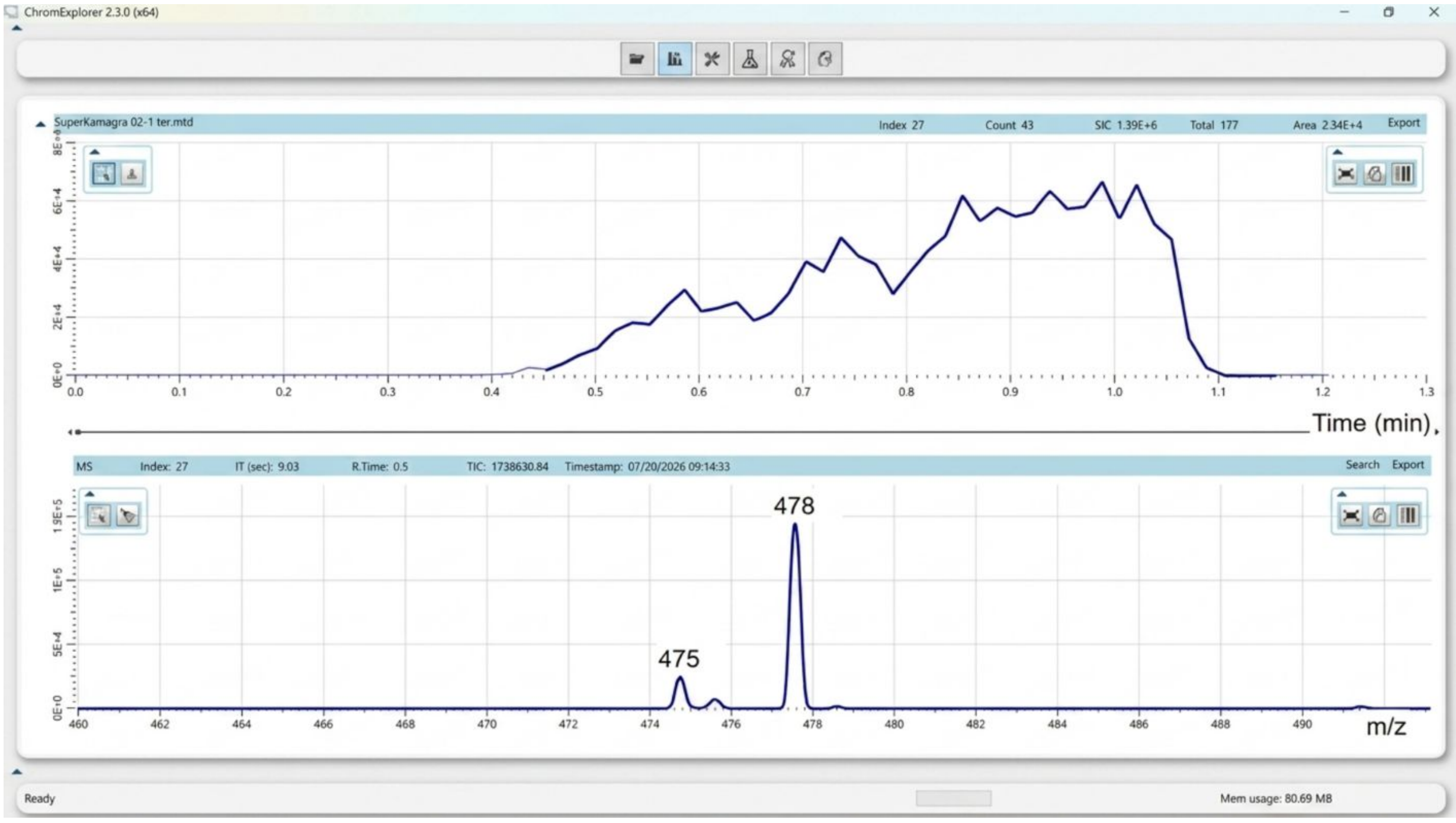

Figure S3 Vardenafil PMS (A) and HRMS (B) fragmentation spectra, C: Proposed fragmentation pattern

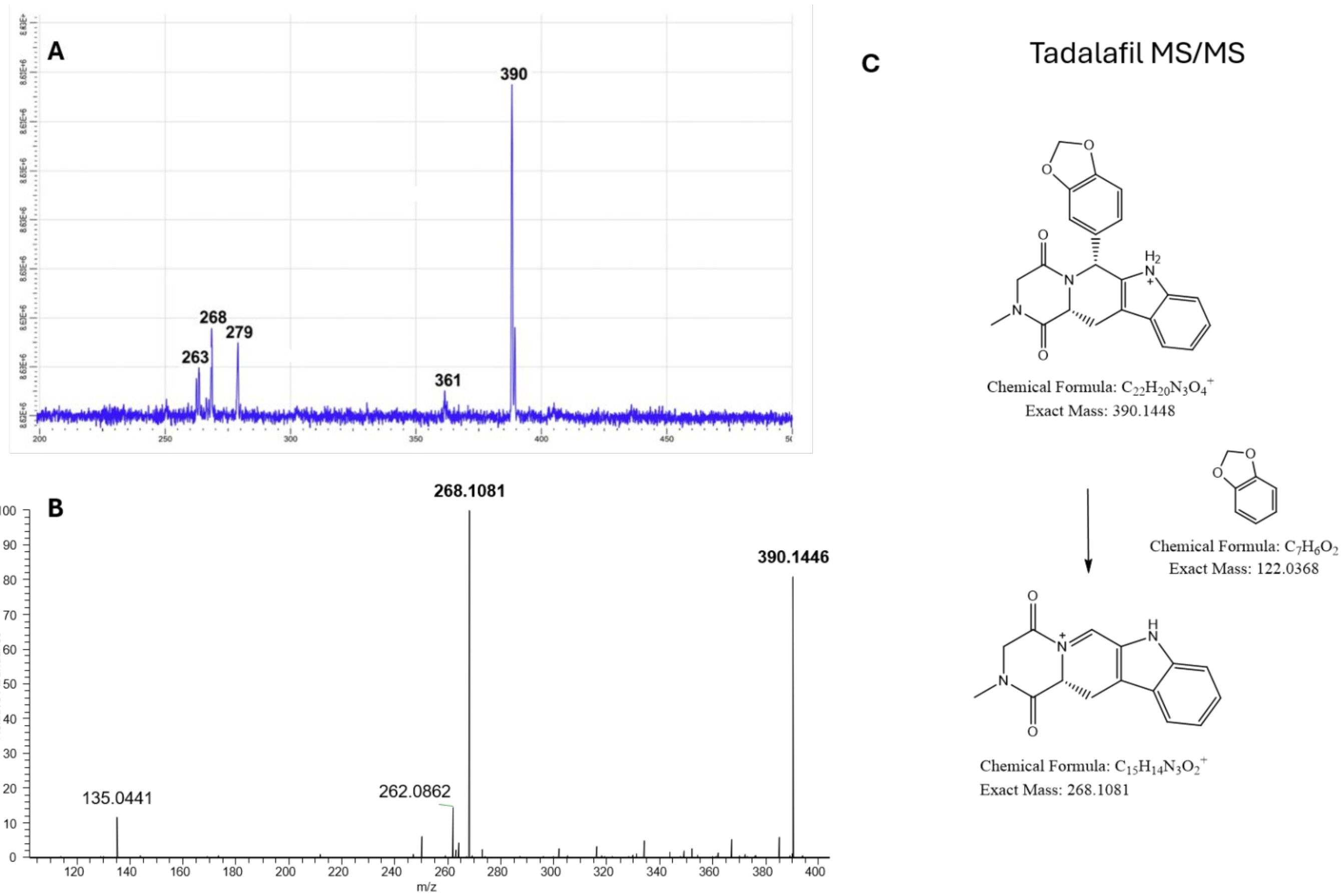

Figure S4 Vardenafil PMS (A) and HRMS (B) fragmentation spectra, C: Proposed fragmentation pattern

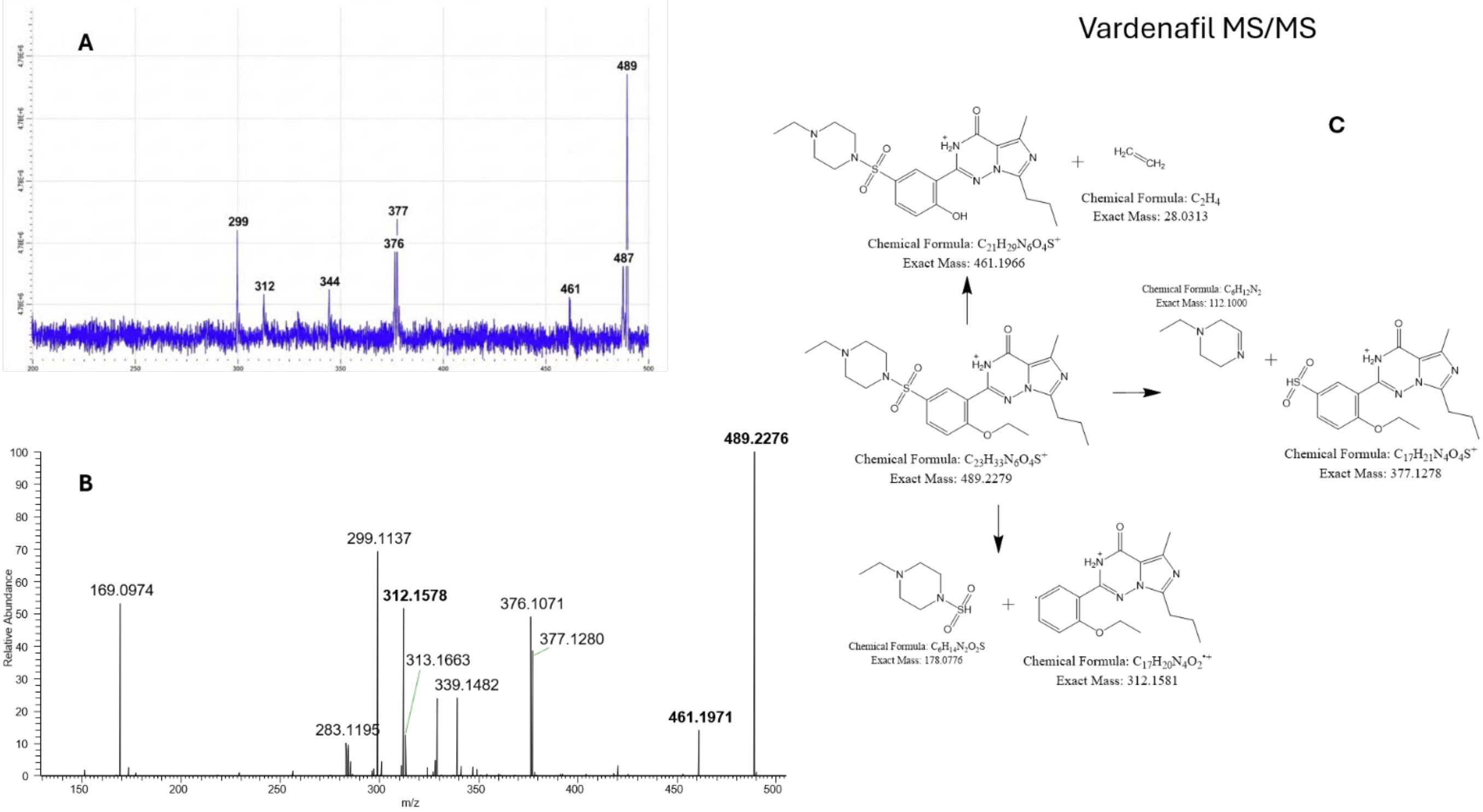

Figure S5 Avanafil PMS (A) and HRMS (B) fragmentation spectra, C: Proposed fragmentation pattern

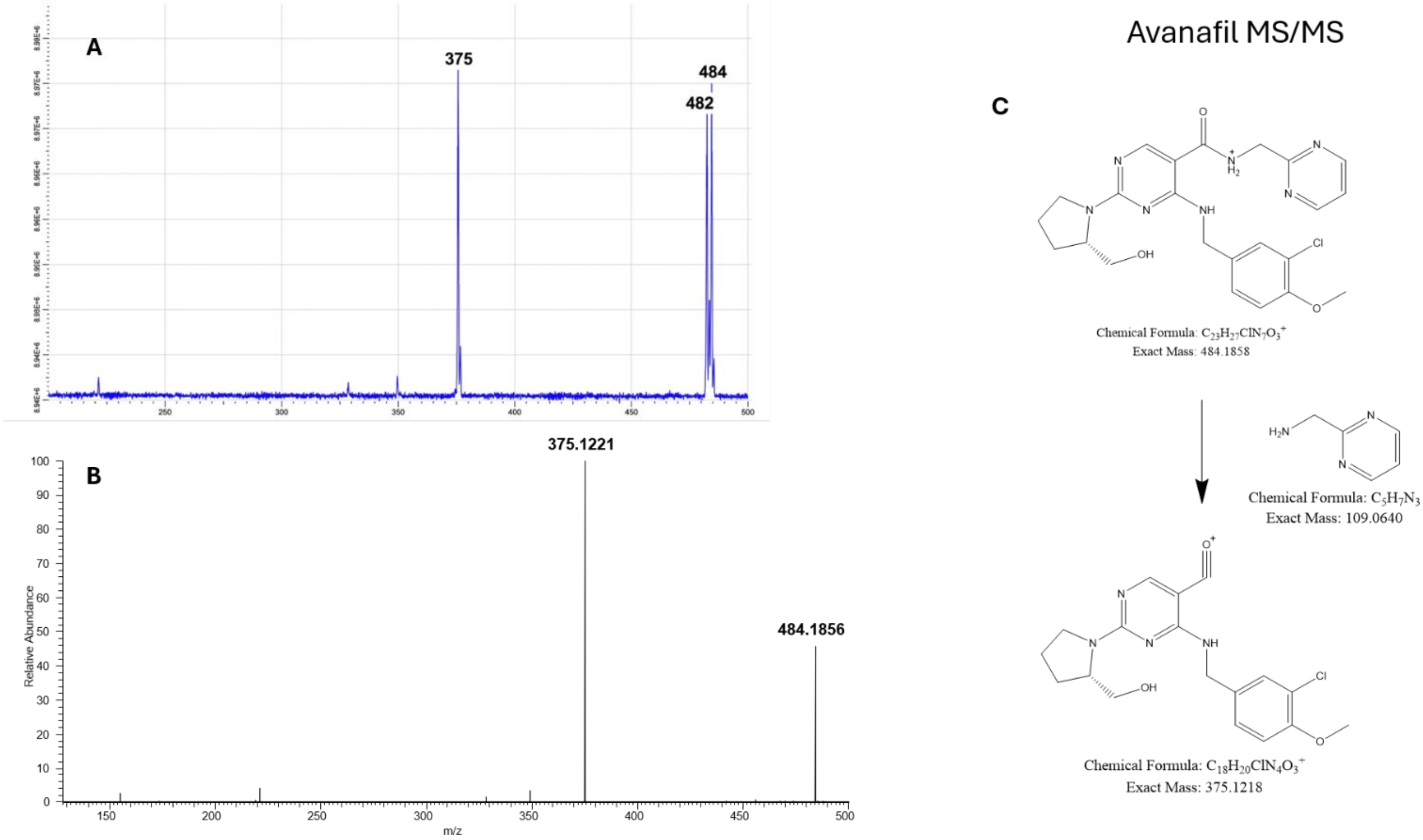

Figure S6 Dapoxetine and Sildenafil protonated ions in PMS (A) PMS (B) and HRMS (C) fragmentation spectra of Dapoxetine, D: Proposed Dapoxetine fragmentation pattern

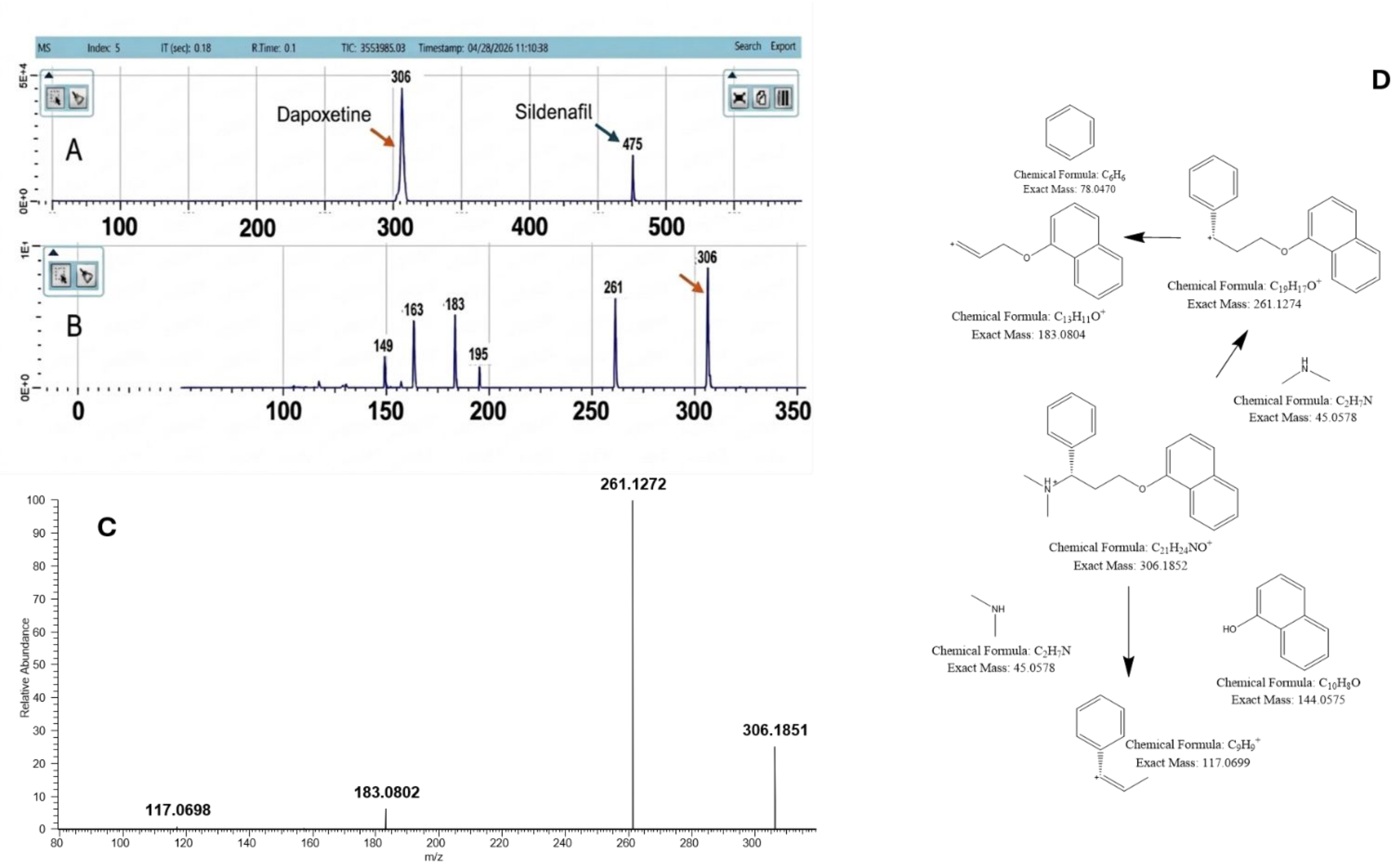

Table S1 Sample description, sample name, form, declared composition and origin.

Sample number	*Sample name*	*Sample form*	*Composition declared*	*Origin*
**1**	Spedra	Tablet	Avanafil 200 mg	Italy, Pharmacy
**2**	Tadalafil	Tablet	Tadalafil 20 mg	
**3**	Levitra	Tablet	Vardenafil 20 mg	
**4**	Viagra	Tablet	Sildenafil 50 mg	
**5**	Joyolgra	Tablet	Sildenafil citrate 100 mg	India Street market
**6**	Vigore 100	Tablet	Sildenafil citrate 100 mg	
**7**	Suhagra 100	Tablet	Sildenafil citrate 100 mg	
**8**	Viro 100	Tablet	Sildenafil citrate 100 mg	
**9**	Cenforce 100	Tablet	Sildenafil citrate 100 mg	
**10**	Caverta	Tablet	Sildenafil citrate 100 mg	
**11**	Suhagra 50	Tablet	Sildenafil citrate 50 mg	
**12**	Apcalis-SX 20	Tablet	Tadalafil 20 mg	
**13**	Super kamagra	Tablet	Sildenafil citrate 100 mg Dapoxetine citrate 60 mg	Thailand Street market
**14**	Lovegra	Tablet	Sildenafil citrate 100 mg	
**15**	Vigas 50000	Spray	none	Italy Police seizure from
**16**	ViaMax Power	Herbal product (tea)	none	

**17**	Big Bang	Cream	none	informal market
**18**	Developpe sex	Cream	none	
**19**	Jekonmo	Herbal product, Liquid	none	
**20**	Manisa	Herbal product, Liquid	none	
**21**	Mudet	Herbal product, Liquid	none	
**22**	Ceedra	Herbal product, Capsule	none	
**23**	Street market blue pills	Tablet	none	Italy
**24**	Street market white pills	Tablet	none	Police seizure

Figure S7: Sample #6 Vigore 100 tablets

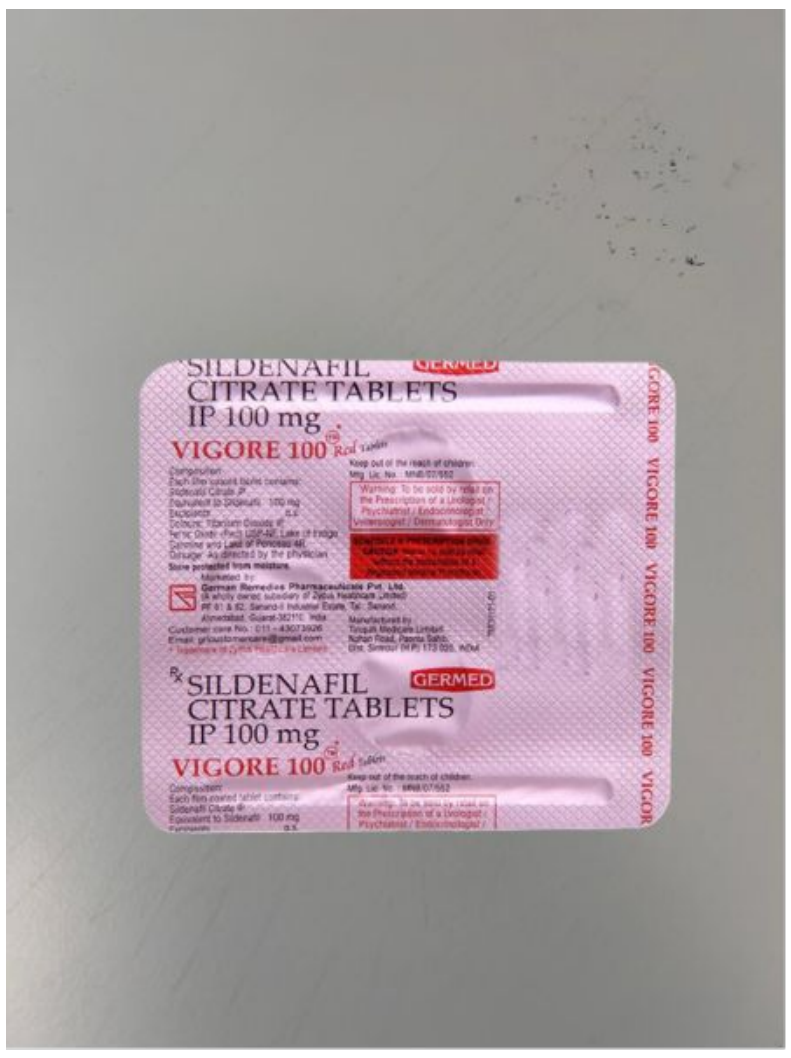

Sample #	Sample Name	Country of origin	Declared API	Detected API
6	Vigore 100	India	Sildenafil citrate 100mg	Sildenafil

Figure S8: Sample #7 Suhagra 100 tablets

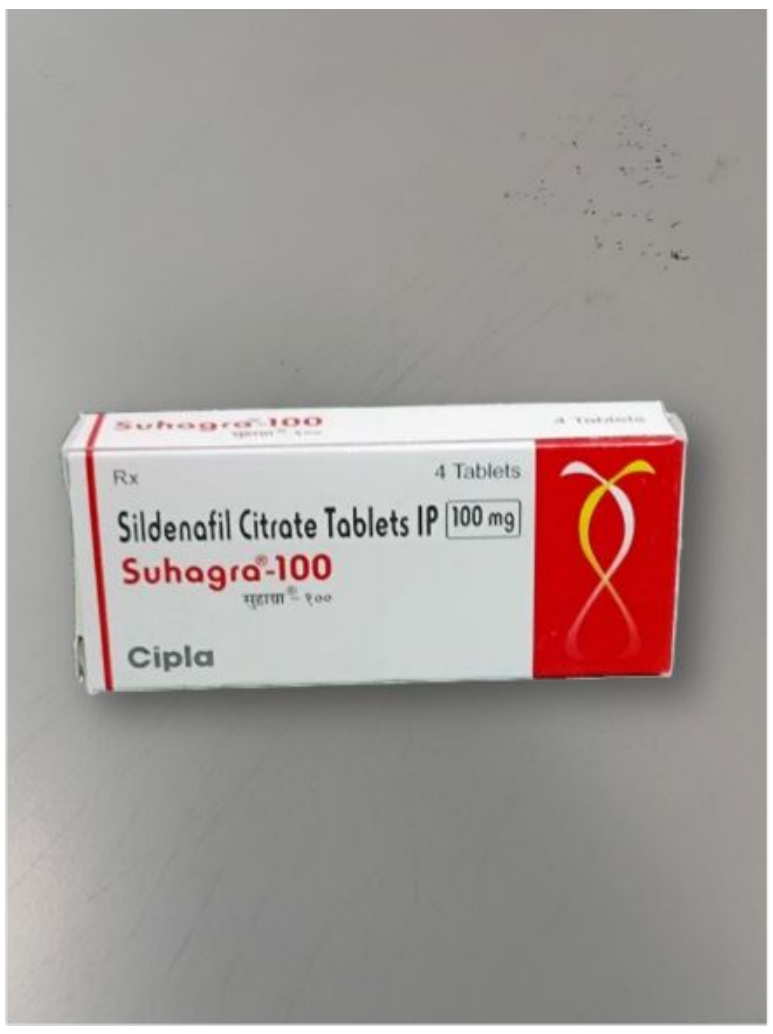

Sample #	Sample Name	Country of origin	Declared API	Detected API
7	Suhagra 100	India	Sildenafil citrate 100mg	Sildenafil

Figure S9: Sample #8 Viro 100 tablets

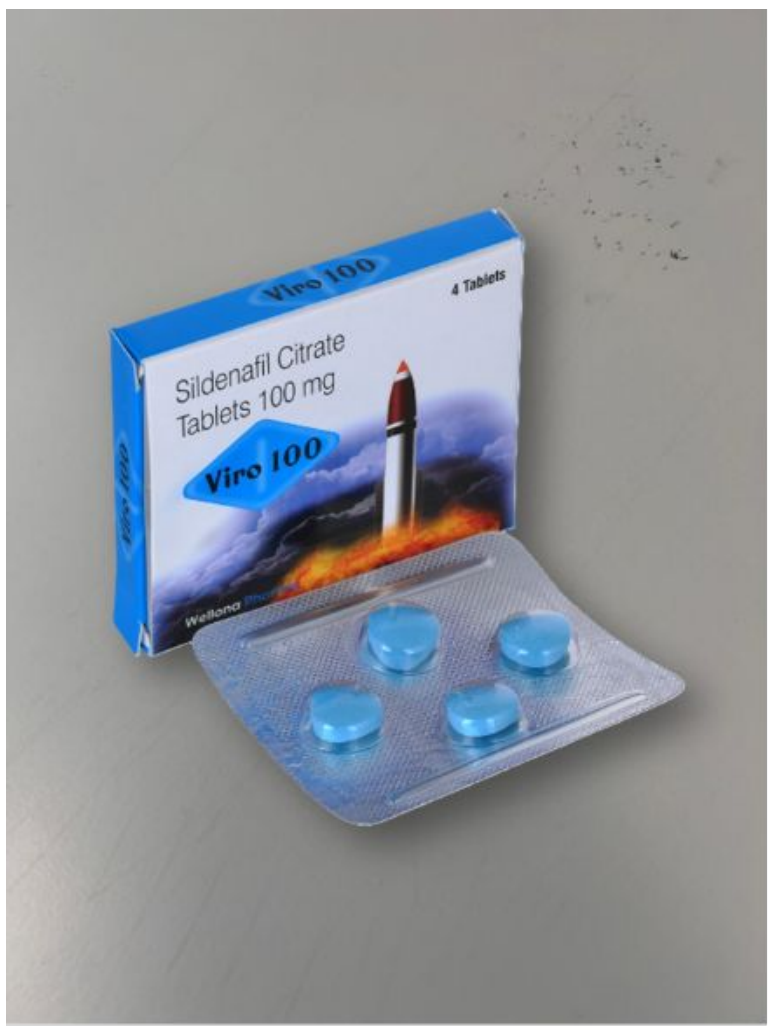

Sample #	Sample Name	Country of origin	Declared API	Detected API
8	Viro 100	India	Sildenafil citrate 100mg	Sildenafil

Figure S10: Sample #9 Caverta 100 tablets

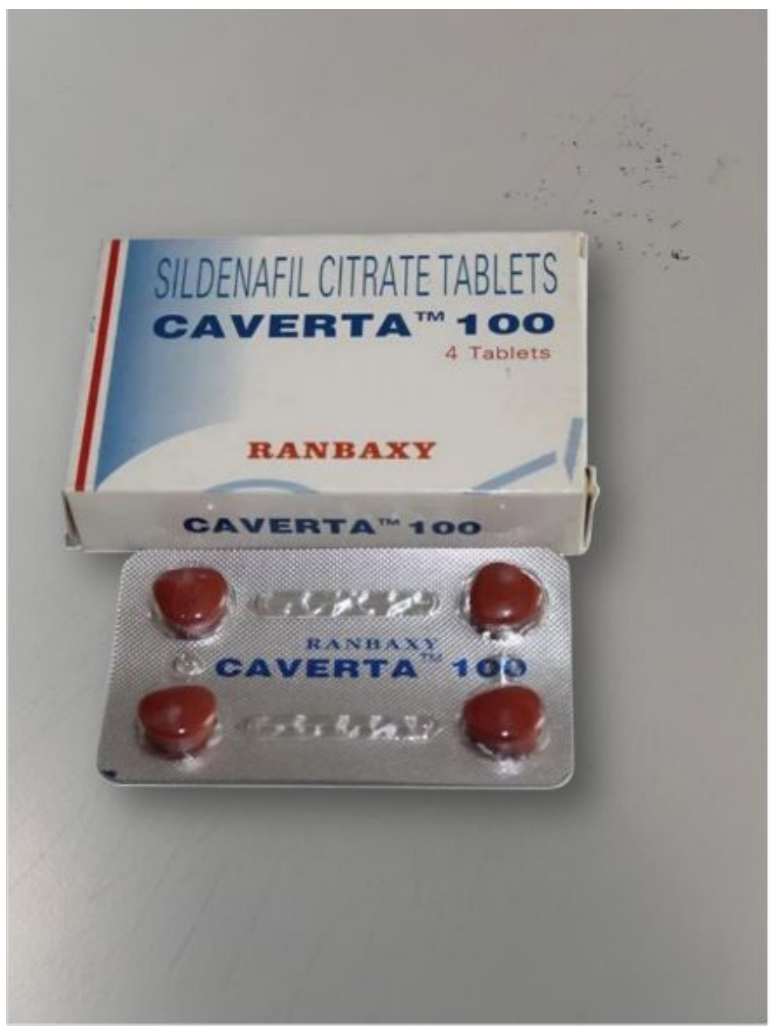

Sample #	Sample Name	Country of origin	Declared API	Detected API
9	Caverta	India	Sildenafil citrate 100mg	Sildenafil

Figure S11: Sample #10 Cenforce 100 tablets

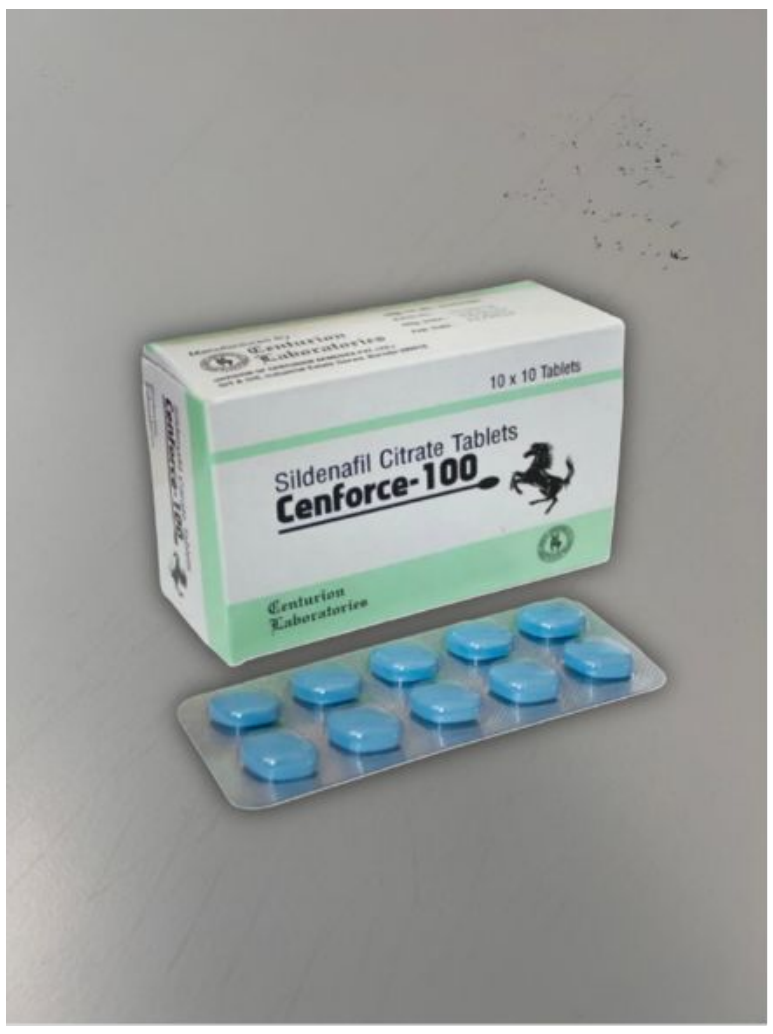

Sample #	Sample Name	Country of origin	Declared API	Detected API
10	Cenforce 100	India	Sildenafil citrate 100mg	Sildenafil

Figure S12: Sample #11 Suhagra 50 tablets

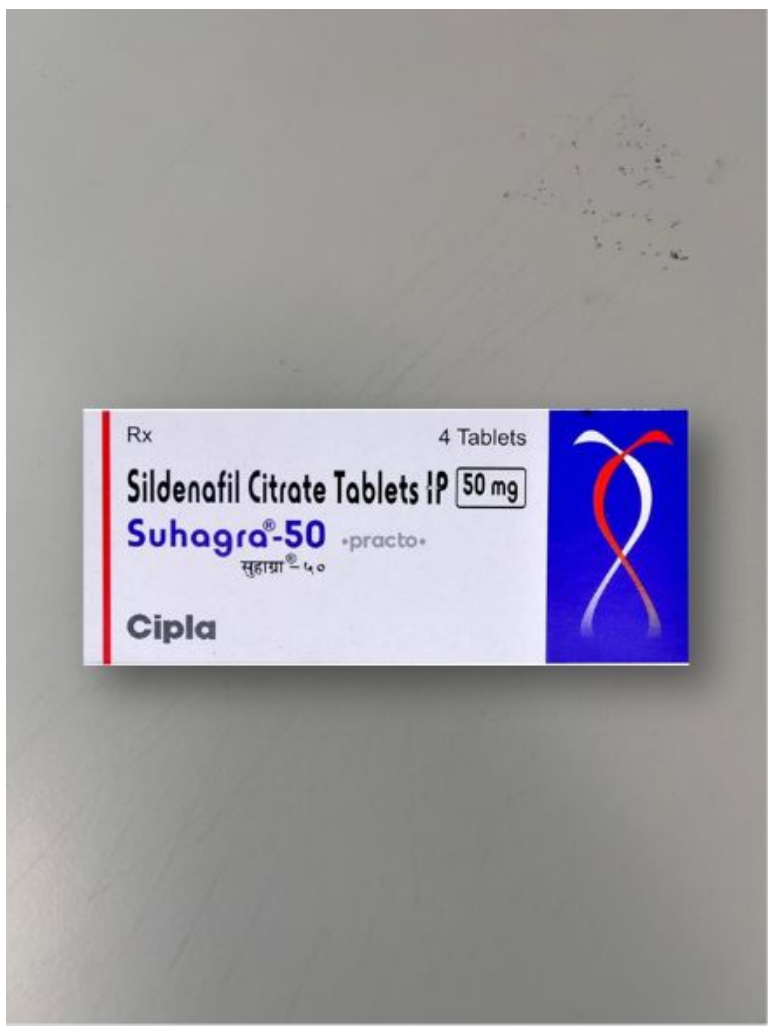

Sample #	Sample Name	Country of origin	Declared API	Detected API
11	Suhagra 50	India	Sildenafil citrate 50 mg	Sildenafil

Figure S13: Sample #12 Apcalis-SX 20 tablets

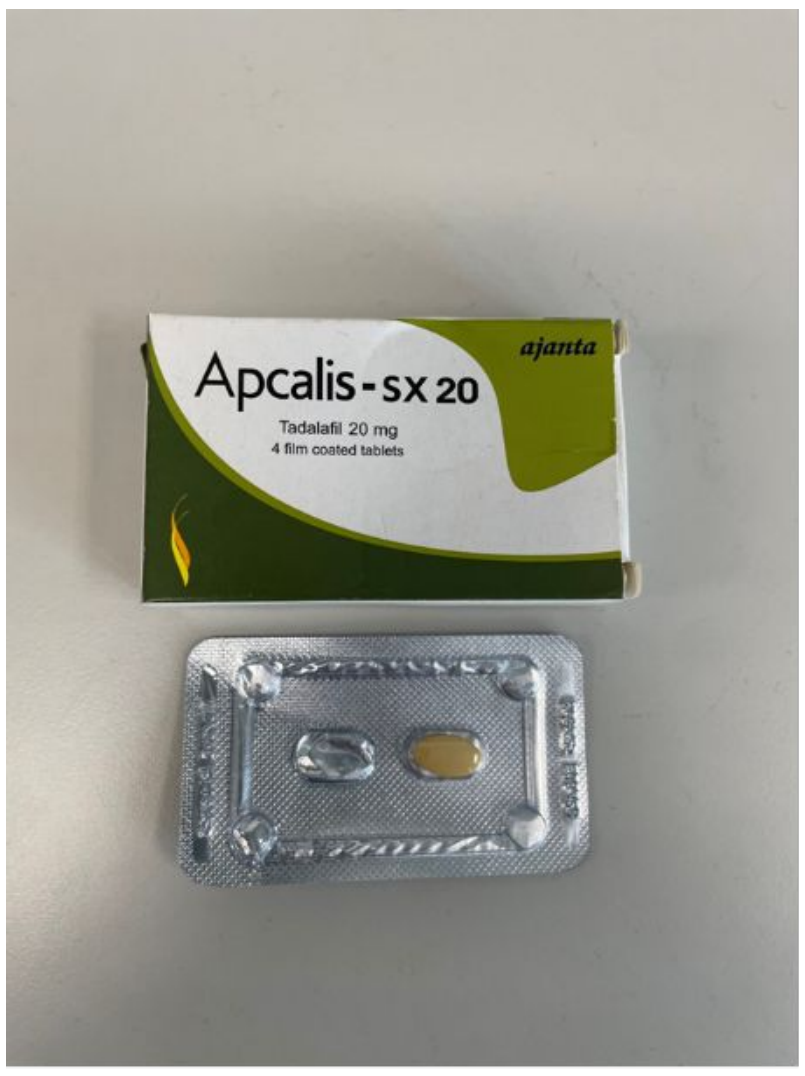

Sample #	Sample Name	Country of origin	Declared API	Detected API
12	Apcalis SX-20	Thailand	Tafalafil 20 mg	Tafalafil

Figure S14: Sample #13 Super Kamagra tablets

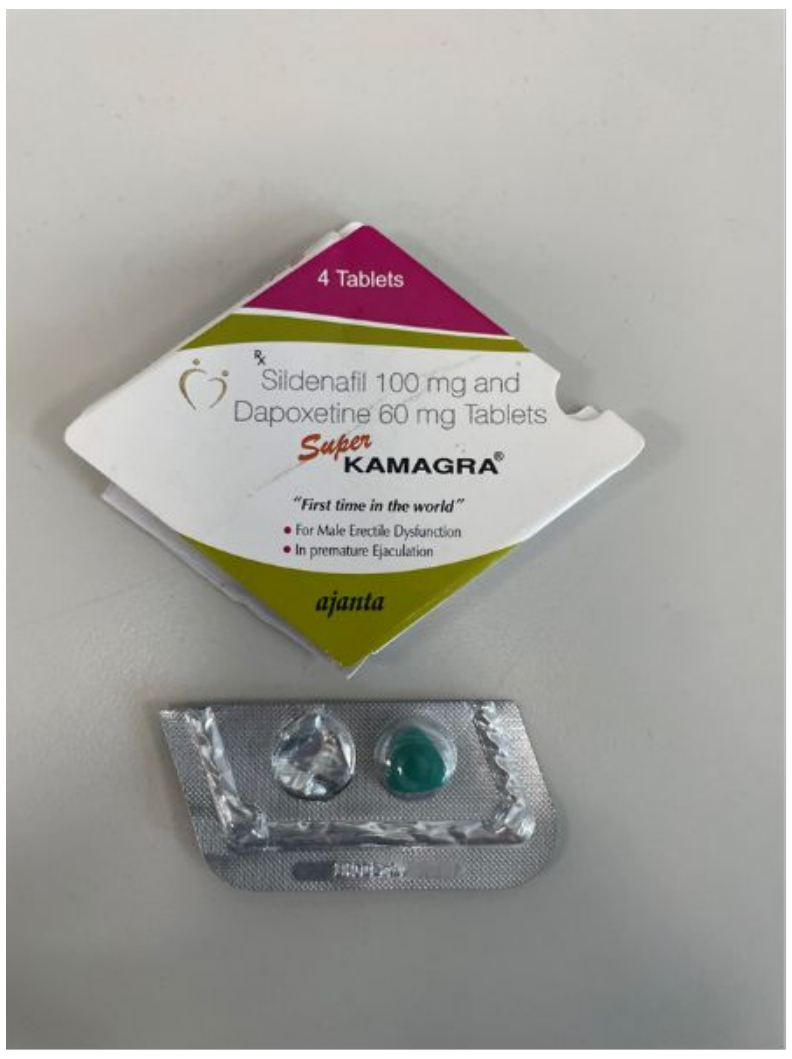

Sample #	Sample Name	Country of origin	Declared API	Detected API
13	Super Kamagra	Thailand	Sildenafil citrate 100 mg<br>Dapoxetine citrate 60 mg	Sildenafil<br>Dapoxetine

Figure S15: Sample #14 Lovegra tablets

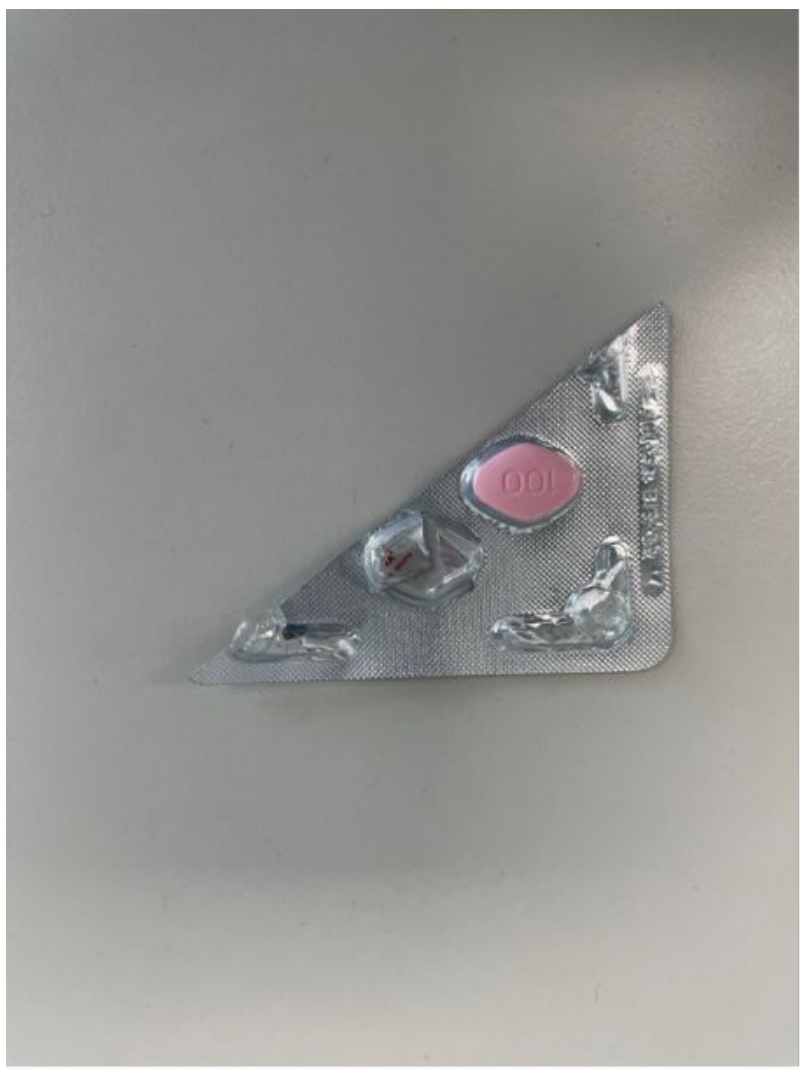

Sample #	Sample Name	Country of origin	Declared API	Detected API
14	Lovegra	Thailand	Sildenafil citrate 100 mg	Sildenafil

Figure S16: Sample #15 Viga 5000 spray

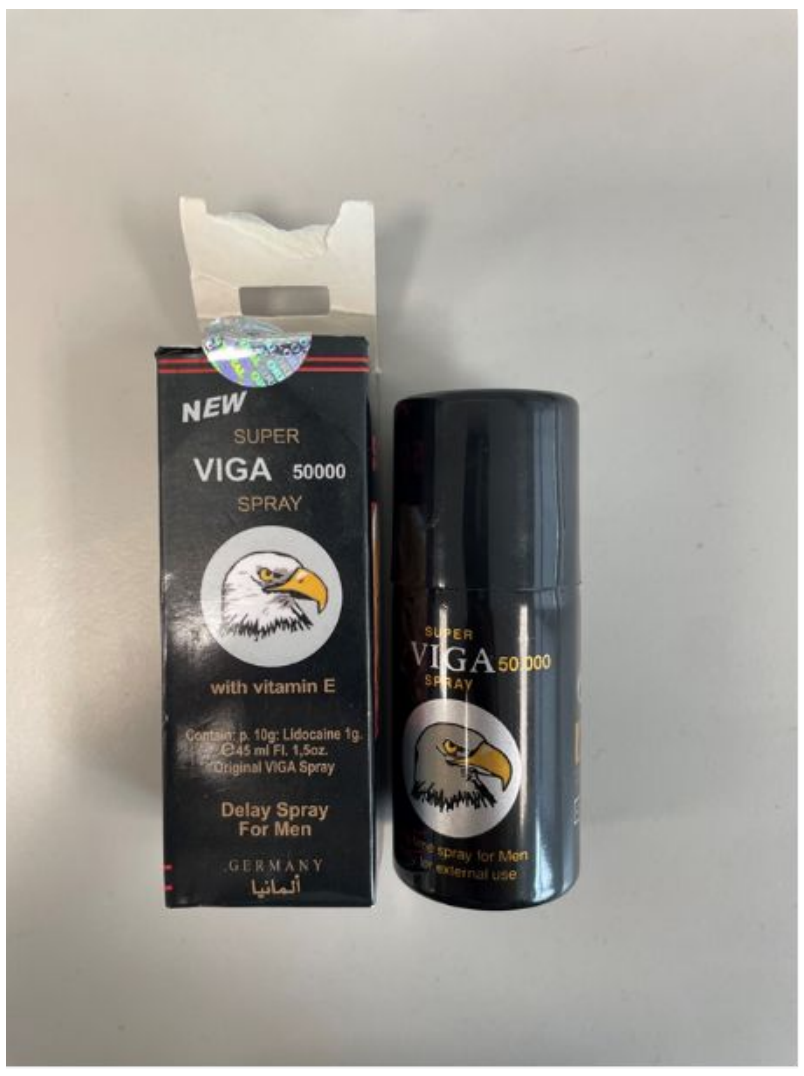

Sample #	Sample Name	Country of origin	Declared API	Detected API
15	Viga 50000	Germany	none	Lidocaine

Figure S17: Sample #16 ViaMax Power coffee (Sexy Coffe)

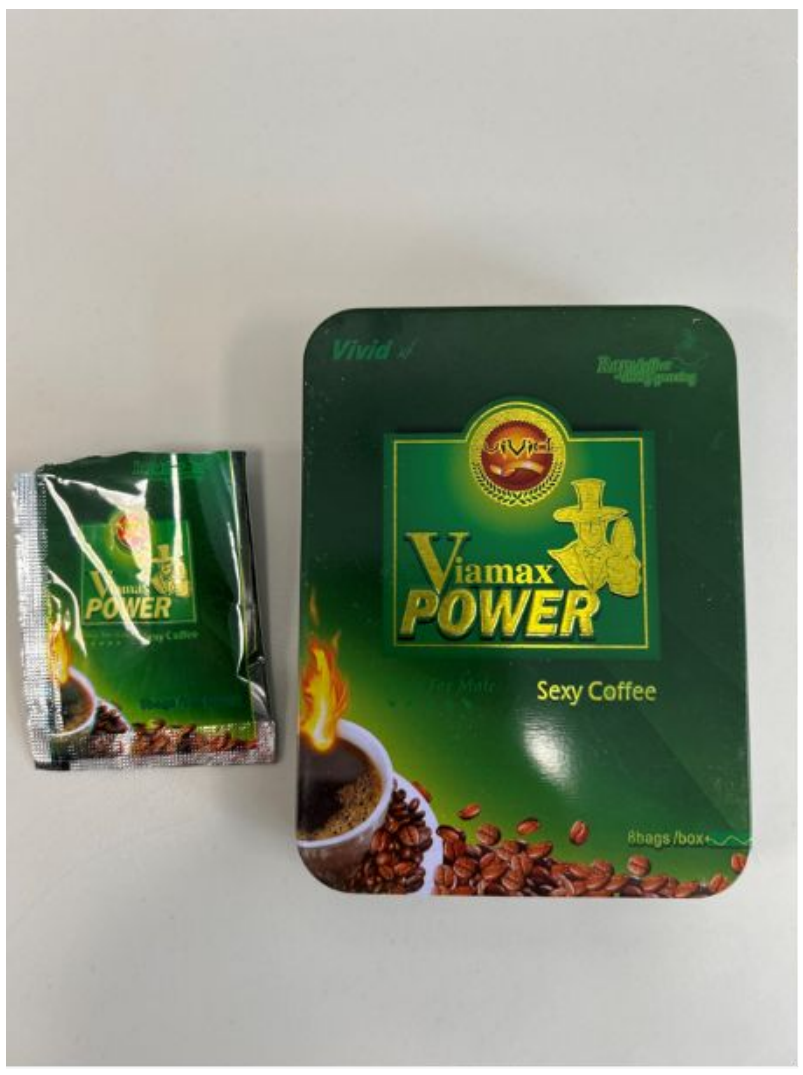

Sample #	Sample Name	Country of origin	Declared API	Detected API
16	ViaMax Power	USA	none	Sildenafil

Figure S18: Sample #17 Big Bang topical cream

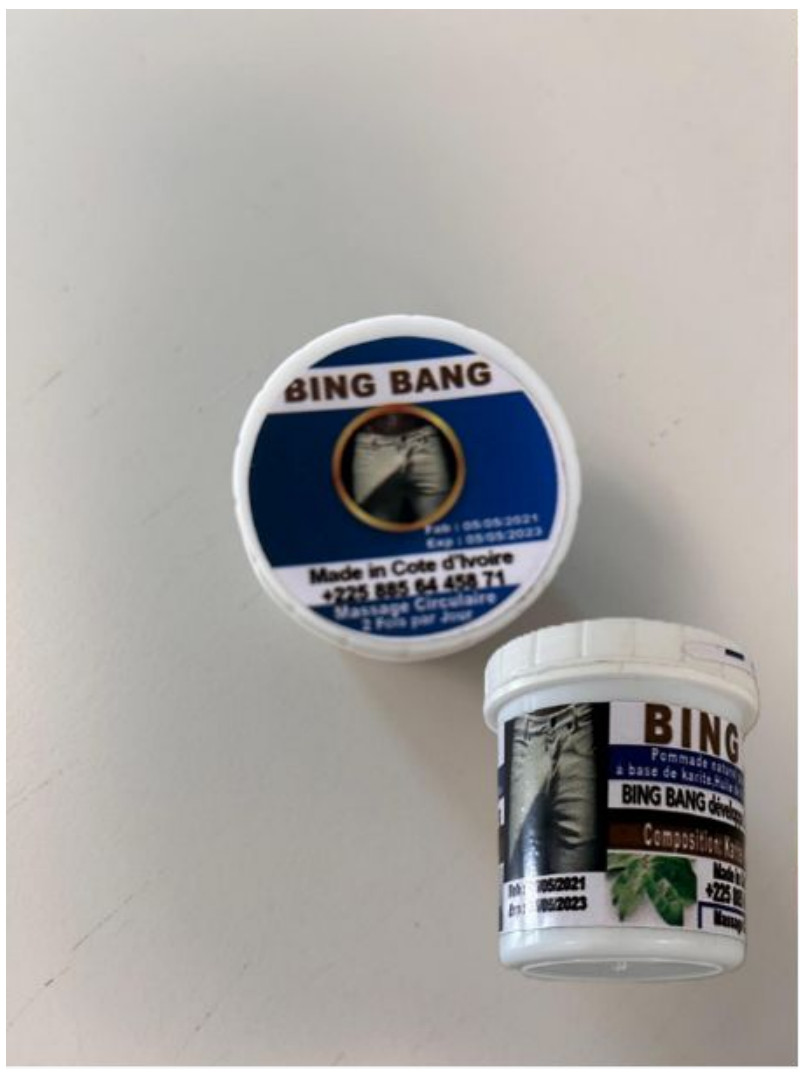

Sample #	Sample Name	Country of origin	Declared API	Detected API
17	Big Bang	Cote d'Ivoire	none	Lidocaine

Figure S19: Sample #18 Developpe SEX topical cream

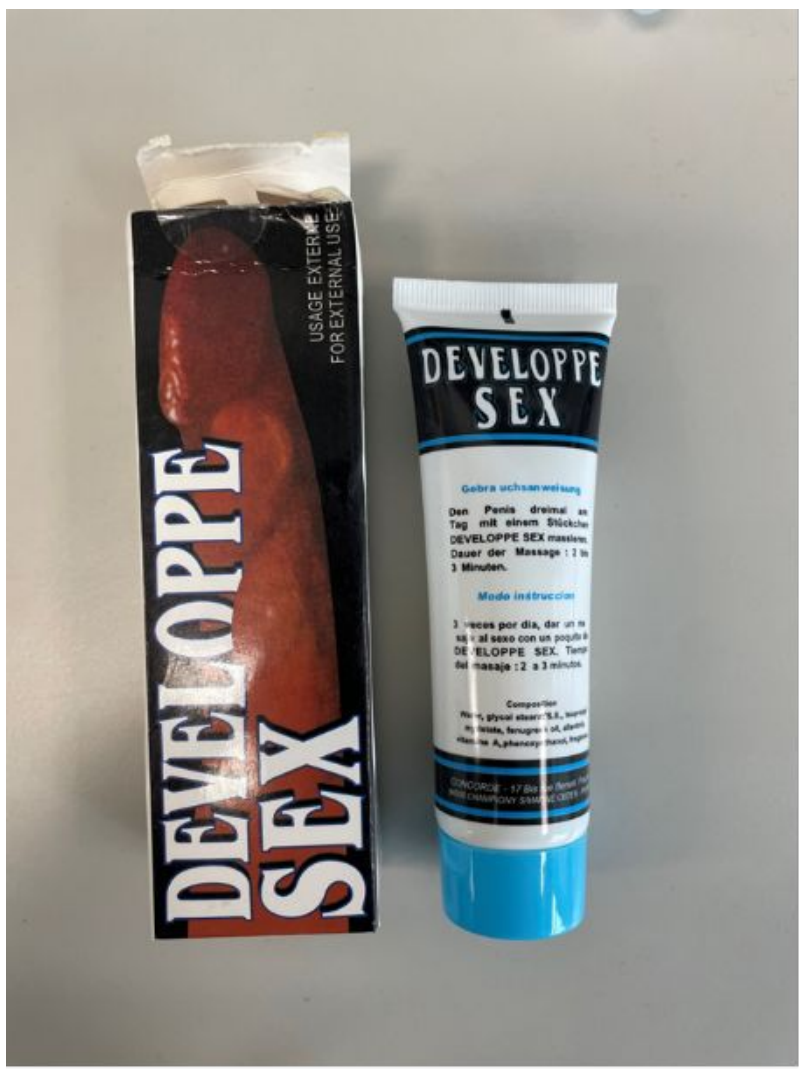

Sample #	Sample Name	Country of origin	Declared API	Detected API
18	Developpe SEX	France	None	Lidocaine

Figure S20: Sample #19 Jekonmo liquid formulation

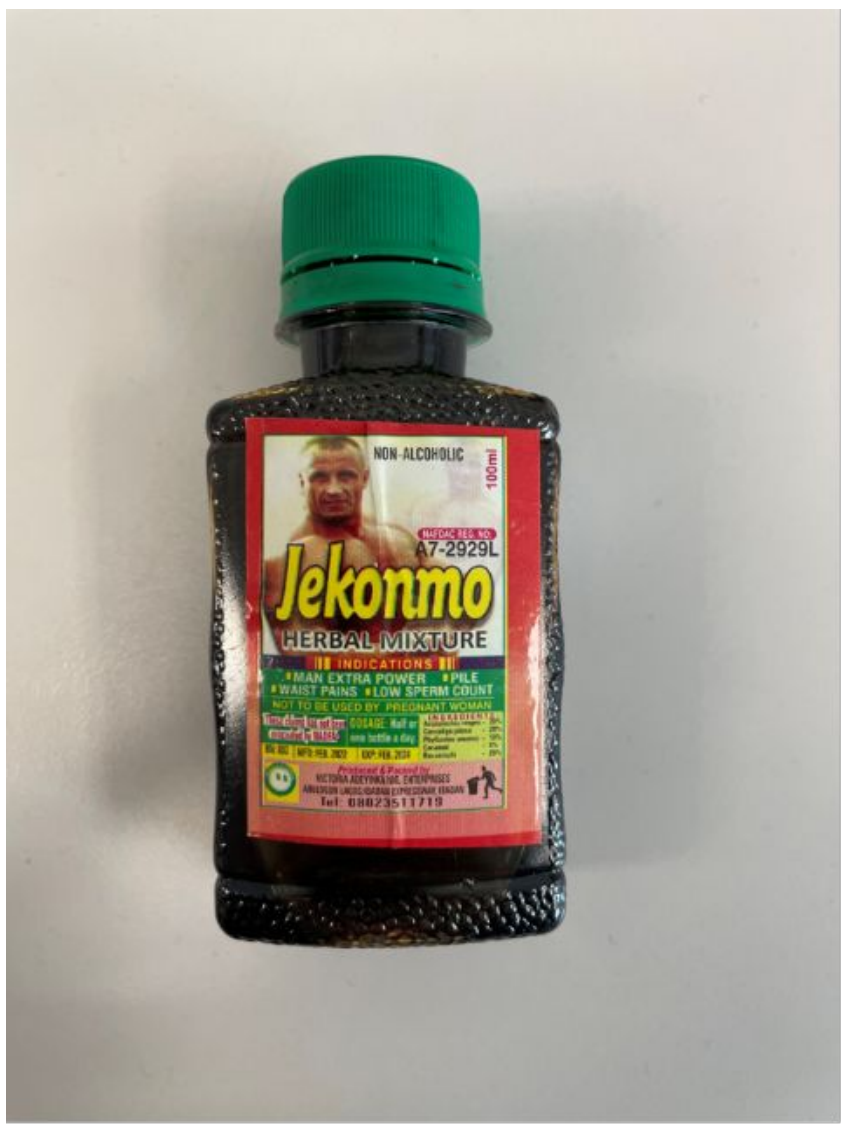

Sample #	Sample Name	Country of origin	Declared API	Detected API
19	Jekonmo	Nigeria	None	Sildenafil

Figure S21: Sample #20 Manisa liquid formulation

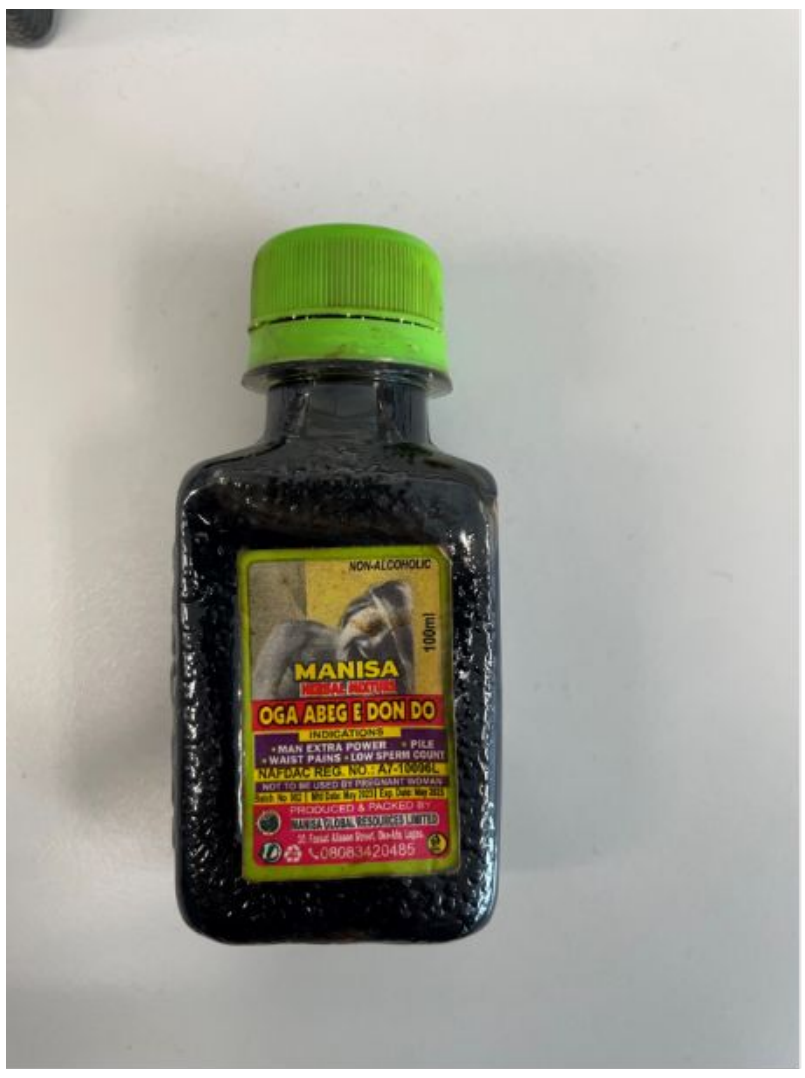

Sample #	Sample Name	Country of origin	Declared API	Detected API
20	Manisa	Nigeria	None	Sildenafil

Figure S22: Sample #21 Mudet liquid formulation

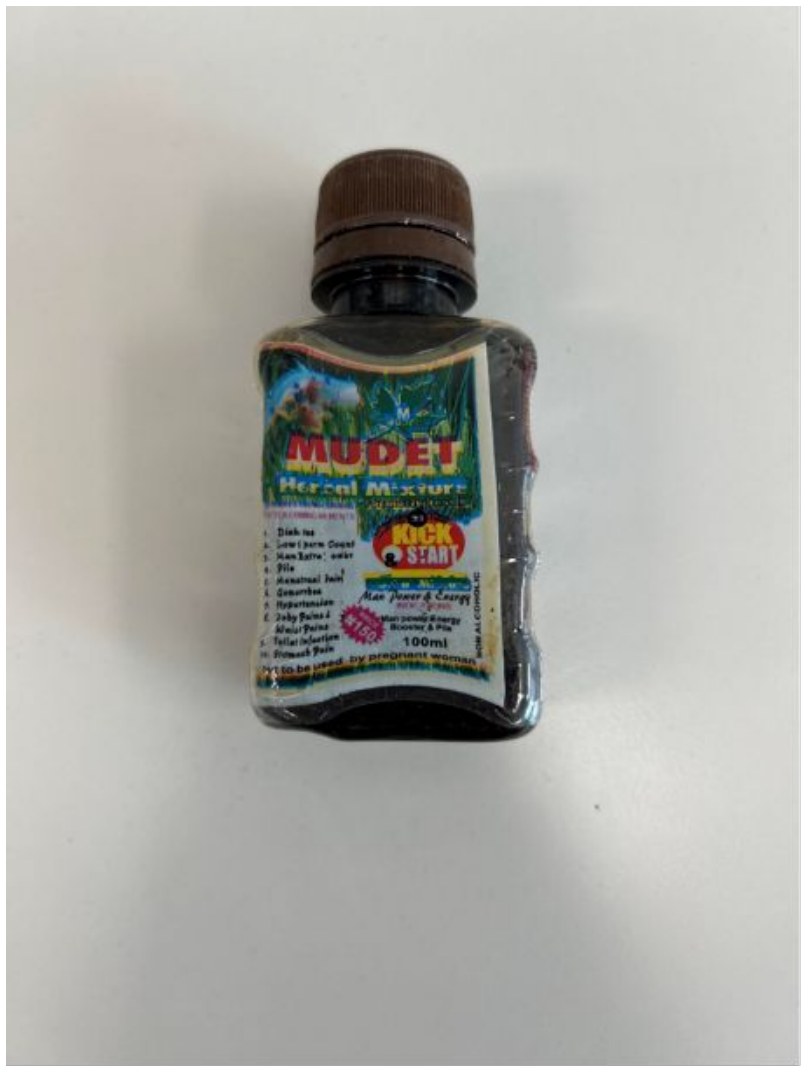

Sample #	Sample Name	Country of origin	Declared API	Detected API
21	Mudet	Nigeria	none	Sildenafil

Figure S23: Sample #22 Ceedra encapsulated formulation

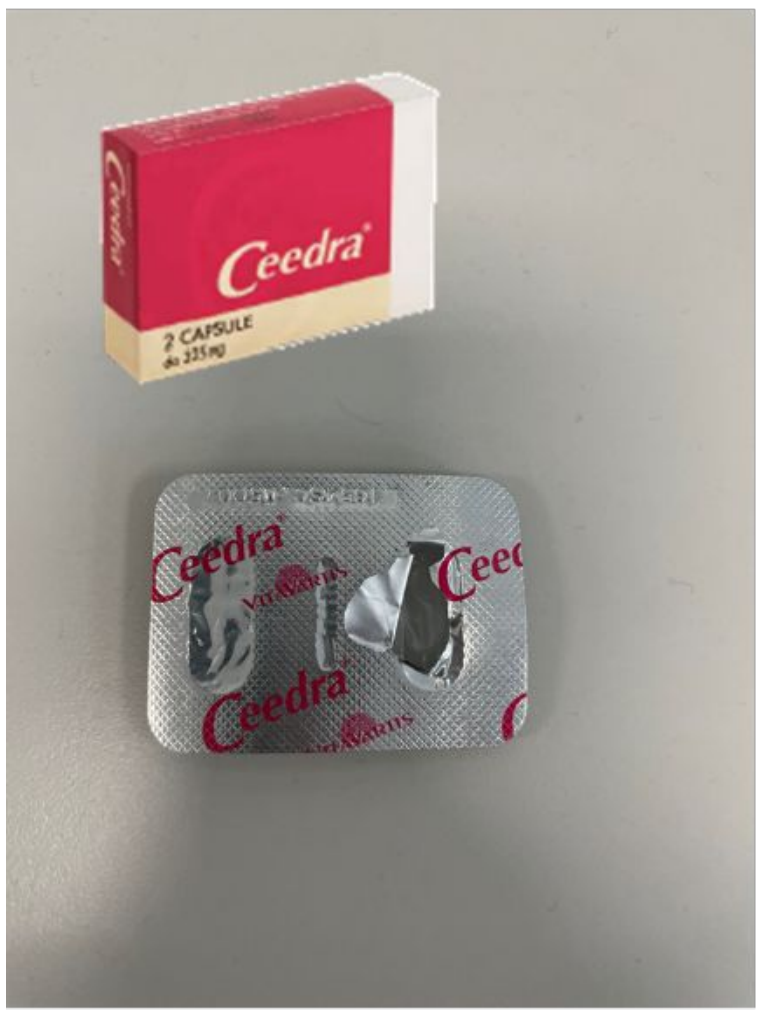

Sample #	Sample Name	Country of origin	Declared API	Detected API
22	Ceedra	Italy	none	Hydroxyhomosildenafil

Figure S24: Sample #23 Street market tablets (blue)

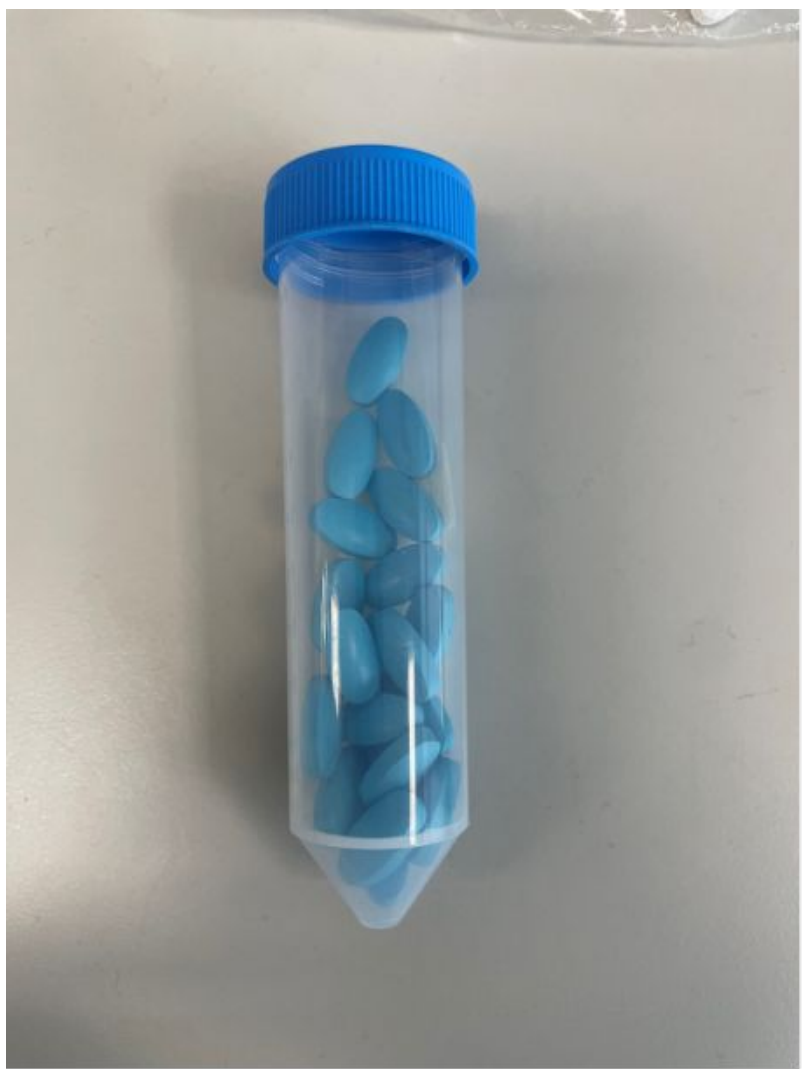

Sample #	Sample Name	Country of origin	Declared API	Detected API
23	Street market blue pills	Italy (Police seizure)	None	None

Figure S25: Sample #24 Street market tablets (white)

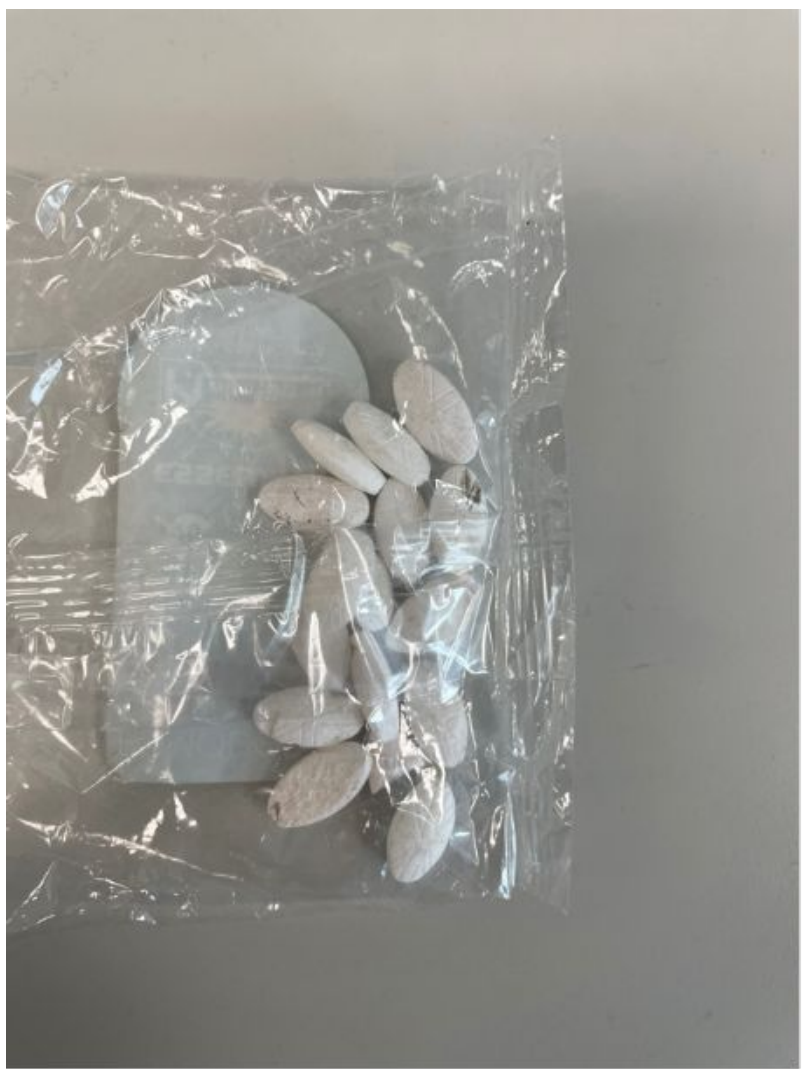

Sample #	Sample Name	Country of origin	Declared API	Detected API
24	Street market white pills	Italy (Police seizure)	None	None
